# Contrasting Diversity and Host Association of Ectomycorrhizal Basidiomycetes versus Root-Associated Ascomycetes in a Dipterocarp Rainforest

**DOI:** 10.1371/journal.pone.0125550

**Published:** 2015-04-17

**Authors:** Hirotoshi Sato, Akifumi S. Tanabe, Hirokazu Toju

**Affiliations:** 1 Center for Ecological Research, Kyoto University 509–3, 2-chome, Hirano, Otsu, Shiga, 520–2113 Japan; 2 National Research Institute of Fisheries Science, Fisheries Research Agency, 2-12-4 Fukuura, Kanazawa-ku, Yokohama, Kanagawa, 236–8648 Japan; 3 Graduate School of Global Environmental Studies, Kyoto University, Yoshida-nihonmatsu-cho, Sakyo-ku, Kyoto, Kyoto, 606–8501, Japan; 4 Graduate School of Human and Environmental Studies, Kyoto University, Yoshida-nihonmatsu-cho, Sakyoku Kyoto, 606–8501 Japan; Institute for Sustainable Plant Protection, C.N.R., ITALY

## Abstract

Root-associated fungi, including ectomycorrhizal and root-endophytic fungi, are among the most diverse and important belowground plant symbionts in dipterocarp rainforests. Our study aimed to reveal the biodiversity, host association, and community structure of ectomycorrhizal Basidiomycota and root-associated Ascomycota (including root-endophytic Ascomycota) in a lowland dipterocarp rainforest in Southeast Asia. The host plant chloroplast ribulose-1,5-bisphosphate carboxylase/oxygenase large subunit (*rbcL*) region and fungal internal transcribed spacer 2 (ITS2) region were sequenced using tag-encoded, massively parallel 454 pyrosequencing to identify host plant and root-associated fungal taxa in root samples. In total, 1245 ascomycetous and 127 putative ectomycorrhizal basidiomycetous taxa were detected from 442 root samples. The putative ectomycorrhizal Basidiomycota were likely to be associated with closely related dipterocarp taxa to greater or lesser extents, whereas host association patterns of the root-associated Ascomycota were much less distinct. The community structure of the putative ectomycorrhizal Basidiomycota was possibly more influenced by host genetic distances than was that of the root-associated Ascomycota. This study also indicated that in dipterocarp rainforests, root-associated Ascomycota were characterized by high biodiversity and indistinct host association patterns, whereas ectomycorrhizal Basidiomycota showed less biodiversity and a strong host phylogenetic preference for dipterocarp trees. Our findings lead to the working hypothesis that root-associated Ascomycota, which might be mainly represented by root-endophytic fungi, have biodiversity hotspots in the tropics, whereas biodiversity of ectomycorrhizal Basidiomycota increases with host genetic diversity.

## Introduction

Plants harbor an array of mutualistic and antagonistic fungi in their root system and rhizosphere, including mycorrhizal, endophytic, litter decomposing, and plant pathogenic fungi [1–3]. Root-associated fungi ubiquitously interact with plants as commensalistic, parasitic and mutualistic symbionts, representing the unseen majority of microbes in root systems and rhizospheres in terrestrial ecosystems [4–6]. They facilitate plant growth and health, maintenance of plant diversity, nutrient cycling, and ecosystem productivity in various forest and grassland habitats [7–11].

Mycorrhizal fungi are one of the most important types of plant symbionts in root systems and the rhizosphere. Among mycorrhizal fungi, ectomycorrhizal (ECM) fungi are the most diverse and one of the most ecologically important guilds, belonging mostly to Basidiomycota and, to a lesser extent, Ascomycota [3, 5, 12]. ECM fungi not only supply soil nutrients (e.g., N and P) to host plants, but also enhance host resistance to drought, disease, and heavy metals in exchange for carbon from host plants [5]. Moreover, ECM mycelial networks that link roots of the same and different tree species have been identified as potential mediators of competition for light and nutrients among plant individuals or species [13, 14]. Although a relatively small number of plant taxa form ECM (e.g., Fagaceae, Betulaceae, Salicaceae, Dipterocarpaceae, Myrtaceae, and Pinaceae; [15]), the importance of ECM fungi is widely recognized because ECM plants dominate various types of temperate and tropical forests [16–20].

Root-endophytic fungi, mainly represented by a polyphyletic assemblage of ascomycetous fungi, are also ubiquitous symbionts in plant root systems. Endophytic fungi colonize plant organs including stems, leaves, and roots, without causing any apparent negative effects to host plants, for at least a part of the host life cycle [21, 22]. The most studied group of root-endophytic fungi are commonly referred to as “dark septate endophytes (DSE),” and include members of the *Phialocephala*–*Acephala* complex, which are common and easily cultured *in vitro* [2, 23, 24]. DSE generally have broad host ranges and are commonly found in terrestrial ecosystems worldwide [24]. Although their physiological effects on host plants remain largely unknown, several studies reported positive effects, including facilitation of host mineral nutrient uptake and enhanced resistance of hosts to soil pathogens [25, 26].

Most studies on the ecology and biodiversity of root-associated fungi have focused on temperate forests in the Northern Hemisphere and on a narrow range of host plant families [25, 27], although it is also worth noting that several recent studies have explored tropical ectomycorrhizal fungal diversity using either sporocarp surveys or molecular techniques [18,28–31]. There is a need to extend our current knowledge about the biodiversity of both ECM and root-endophytic fungi in tropical forests for a better understanding of the overall global pattern of biodiversity of root-associated fungi. ECM as well as arbuscular mycorrhizal fungi are increasingly recognized as ecologically important components in tropical ecosystems [16, 18, 32, 33], particularly in the paleotropical rainforests of Southeast Asia, which are characterized by a high abundance of ECM trees in the Dipterocarpaceae [34]. Several recent studies have suggested that biodiversity of ECM fungi was greater in temperate than in tropical regions [35, 36], in clear contrast to the general latitudinal gradient of biodiversity found in plants and insects (i.e., increasing species richness at lower latitudes, [37]). On the other hand, tropical forests were postulated as putative biodiversity hotspots for endophytic fungi [38, 39] based on the diversity of foliar endophytic fungi. Because of limited availability of data for biodiversity of root-associated fungi in the tropics, latitudinal diversity patterns of these fungi remain unclear.

Lack of knowledge of root-associated fungal diversity in the tropics also impedes our understanding of the level of host preference of these fungi. ECM fungi have been shown to exhibit strong host preference at host genus or family levels in many studies [20, 30,40,41] (however, also see [33]), whereas root-endophytic fungi appeared to show low [2, 21, 24] or moderate levels of host preference [42, 43]. However, little is known about host preference of ECM and root-endophytic fungi in tropical forests, particularly in dipterocarp rainforests, despite the fact that specificity of host–fungus associations in species-rich tropical forests has invaluable implications for the understanding of fungal biodiversity [8].

In this study, we examined the community of putative ECM Basidiomycota and root-associated Ascomycota in lowland, mixed-dipterocarp forests in the Lambir Hills National Park, Sarawak, Borneo. We used massively parallel 454 pyrosequencing to evaluate the diversity and host range of both ECM Basidiomycota and root-associated Ascomycota co-occurring with Dipterocarpaceae and other plant groups. We hypothesized that the diversity of ECM Basidiomycota would be moderate because their diversity was constrained by the genus- or family-level phylogenetic diversity of host plants. In contrast, the diversity of root-associated Ascomycota, which might be mainly represented by root-endophytic Ascomycota, was predicted to be considerably higher in the tropical rainforest, following the general latitudinal biodiversity gradient [37]. To examine this hypothesis in more detail, we tested whether host genetic distance correlated with fungal community composition by evaluating the decay of root-associated fungal community similarity with increasing levels of genetic distance between host plants.

## Materials and Methods

### Study area and sampling methods

The sampling of plant roots was conducted in the Lambir Hills National Park, (N4°12', E114°02', 130–150 m a.s.l.), located in northern Borneo in the Malaysian state of Sarawak. The climate is aseasonal with an average annual rainfall of approximately 3000 mm and mean monthly rainfall of >100 mm. The area consists of approximately 6500 ha of primarily lowland, mixed tropical forest. The forest in the study site was intact and mature with Dipterocarpaceae dominant in the canopy layer and Euphorbiaceae, Burseraceae, and Myristicaceae being dominant in the lower layers. The Dipterocarpaceae are known to be ectomycorrhizal [34]. Other potential ectomycorrhizal hosts occurred in the plot but were relatively rare (e.g., Myrtaceae, Leguminosae, and Fagaceae). The rocks are sedimentary, composed of alternating layers of sandstone and shale.

Samples were collected from February 2–5, 2012, and consisted of 1-cm segments of terminal roots from the upper part of the A horizon of the sandstone-derived soil at 1-m intervals along a 599-m linear transect (i.e., one sample from each of 600 sampling positions). The linear transect was entirely along a ridge where the vegetation was left intact and potential ECM trees (i.e., Dipterocarpaceae) were relatively dominant. Terminal roots were sampled regardless of their morphological features, and thus included roots were potentially colonized by ECM, root endophytes, or other root-associated fungi (e.g., pathogens and saprotrophs). The root samples were carefully washed free of soil and dried using silica gel.

Our study was conducted in accordance with a Memorandum of Understanding signed between the Sarawak Forestry Corporation and the Japan Research Consortium for Tropical Forests in Sarawak in November 2005. The field studies did not target endangered or protected species.

### Molecular analyses

Root samples were pulverised using a TissueLyser II (QIAGEN) by shaking with 4-mm zirconium balls 20 times per second for 3 min. Plant and fungal DNA was extracted from each root sample using a previously-described cetyl trimethyl ammonium bromide (CTAB) method [44].

The host plant chloroplast ribulose-1,5-bisphosphate carboxylase/oxygenase (*rbcL*) large subunit region and fungal nuclear ribosomal internal transcribed spacer 2 (ITS2) region were sequenced using tag-encoded, massively parallel pyrosequencing. For each root sample, a 0.5-kb *rbcL* gene fragment was amplified using the forward primer rbcL_F3 [45] fused with the 454 pyrosequencing Adaptor A (5′-CCA TCT CAT CCC TGC GTG TCT CCG ACT CAG-3′) and an 8-mer molecular ID sequence [46] unique for each sample, and the reverse primer rbcL_R4 [45] fused with the 454 Adaptor B (5′-CCT ATC CCC TGT GTG CCT TGG CAG TCT CAG-3′). For the analysis of fungal ITS sequences, the ITS2 region was PCR-amplified with a nested PCR method, which helps prevent a biased estimate of the fungal community composition due to uneven amplification among samples. The entire ITS region and a partial ribosomal large subunit region were amplified using the fungus-specific high-coverage primer ITS1F_KYO2 [47] and the universal primer LR3 (http://www.biology.duke.edu/fungi/mycolab/primers.htm). The second PCR was conducted with a universal primer ITS3_KYO2 [47] fused with the 454 Adaptor A and each sample-specific molecular ID, and the reverse universal primer LR_KYO1b [45] fused with the 454 Adaptor B. The PCR was conducted using conditions detailed elsewhere [45].

The *rbcL* and second-round ITS amplicons were subjected to pyrosequencing. The PCR products were pooled and then purified using ExoSAP-IT (GE Healthcare, UK) or the QIAquick PCR Purification Kit (QIAGEN). Pyrosequencing was performed with a 454 GS Junior sequencer (Roche) according to the manufacturer’s instructions. To increase the number of ITS reads per sample, two independent runs were performed.

### Bioinformatic analyses

For the pyrosequencing reads, low-quality 3′ tails were trimmed to a minimum quality value of 27. After trimming, *rbcL* and ITS reads shorter than 150 bp excluding the forward primer and molecular ID positions were discarded. The *rbcL* and ITS reads were recognized by primer position sequences and analyzed separately. For each gene, pyrosequencing reads were sorted based on the sample-specific molecular IDs. Molecular IDs and forward primer sequences were removed before the assembly process. The reads within individual samples were assembled using a cut-off similarity of 97%, with the assembler Assams v0.1.2012.05.24 [48], a highly parallelized extension of the Minimus assembler [49]. This within-sample assembling helped to avoid overestimating operational taxonomic unit (OTU) richness that could be caused by pyrosequencing errors. For each sample, potentially chimeric sequences were then eliminated by UCHIME v4.2.40 [50] with a minimum score to report chimeras of 0.1.

The within-sample contigs that passed the chimera removal process were assembled across samples by Assams, and the resulting consensus sequences represented molecular OTUs, S1 Dataset. The *rbcL* contigs were subjected to assembling at each of the four cut-off similarities of 99.8%, 99%, 98%, and 97%. There were a number of samples that yielded more than one *rbcL* contig, possibly due to the presence of chimeric sequences. The highly dominant *rbcL* contigs in samples likely represented sequences of host plant species, and thus the *rbcL* contigs with >95% of the sample total reads were designated as representative sequences of host plant species. In the analysis of the fungal ITS2 region, the minimum cut-off similarity for the inter-sample assembling process was set at 95%. The results of the statistical analysis at a cut-off similarity of 97% were qualitatively similar to those at 95% (data not shown). To remove sequences with high proportions of pyrosequencing errors, singletons were excluded in the analyses of fungal data.

The taxonomic identity of the OTUs was assigned based on the barcode sequences of fungi (ITS2 region) and plants *(rbcL*) using Claident v0.1.2012.05.21 [51] (available at website: http://www.fifthdimension.jp/products/claident/), which allowed automated execution of the lowest common ancestor algorithm [52] integrating BLAST+ [53] and NCBI taxonomy-based sequence identification engines. The query-centric auto-k-nearest-neighbor (QCauto) method [51] in Claident was used for the molecular identification of OTUs, with local BLAST databases prepared based on the “nt” database downloaded from the NCBI ftp server (http://www.ncbi.nlm.nih.gov/Ftp/) as of May 11, 2012. Claident aids in the reliable identification of fungi detected in environmental samples based on a theoretically explicit criterion [51] and can be used for the taxonomic assignment of both ECM and root-endophytic fungi [54, 55]. Based on the resulting genus and family level taxonomic information output, putatively ECM basidiomycetous OTUs were identified based on published reviews [3, 5], S6 Dataset.

### Data matrix and accumulation curve of OTU richness

The sequencing reads from respective fungal OTUs were recorded for each of the 546 samples from which *rbcL* sequences were successfully obtained and arranged into a matrix in which the rows and columns represent sample IDs and fungal OTUs, respectively (S2 Dataset). To reduce variance in α-diversity among samples that resulted from variance in sequencing effort, the dataset was rarefied to a depth of 100 reads (‘rrarefy’ function in vegan v.2.0–3 package of R; [56]), and 104 samples with lower sequencing depths (less than 100 reads) were removed, S2 Dataset. The remaining samples were used to produce a binary presence/absence matrix for respective fungal OTUs in each sample (sample-level matrix, S3 Dataset). Sample-based OTU accumulation curves were then calculated based on the sample-level matrix for ascomycetous taxa, basidiomycetous taxa, and ECM basidiomycetous taxa using the ‘specaccum’ command in the vegan package.

For the following analyses of host associations, the sample-level matrix was converted into four plant taxa x fungal OTU matrices (hereafter, plant x fungal matrices), with host plants clustered at a cut-off *rbcL* sequence similarity of 99.8%, 99%, 98%, or 97%, S4 Dataset. In each plant x fungal matrix, rows represent plant taxa and columns represent fungal OTUs, with cell entries *y*_i,j_ as integers representing the number of samples in which fungal OTU *j* was present in plant OTU *i*.

### Statistical analyses for assessing host association

Host association of root-associated fungi was evaluated by assessing whether the observed number of root samples of plant OTU *i* colonized by fungal OTU *j* deviated from the number that would be expected under the assumption that fungi randomly associated with host plants. While each plant x fungal matrix should reflect the relative degree of host plant association for individual fungal OTUs, potential biases attributable to inter-sample variation in sampling efforts were also included in this matrix. To address such potential biases, we used a Bayesian analysis implementing a Dirichlet-multinomial model to control for the potential effect of uneven sampling effort.

For each of the four plant x fungal matrices, let *y*_i,j_ be the observed number of associations between fungal OTU *i* and plant OTU *j*. The model assumes that *y*_i,j_ follows a multinomial distribution:

$$Multi(y_{i,j}|\pi_{i,j})=\left(\begin{array}{c} N_{i}\\ y_{\text{i,1}},\cdots y_{\text{i,j}} \end{array}\right)\prod_{j=1}^{J}\pi_{\text{i,j}}{}^{y_{\text{i,j}}}$$

where *N*_i_ is the total number of root samples in which fungal OTU *i* was detected, and *π*_i,j_ is the proportion in which fungal OTU *i* was associated with plant OTU *j*. The variable *π*_i,j_ is described in the Dirichlet distribution as follows:

$$\begin{array}{l} Dir(\pi_{i,j}|\alpha_{i,j})=\frac{\Gamma (\sum_{j=1}^{J}\alpha_{i,j})}{\Gamma (\alpha_{i,1})\ldots \Gamma (\alpha_{i,j})}\prod_{j=1}^{J}\pi_{i,j}{}^{\alpha_{i,j}-1},\\ \alpha_{\text{i,j}}=\frac{T_{j}N_{i}}{\sum T_{j}}, \end{array}$$

where *α*_i,j_ is a parameter of the Dirichlet distribution that reflects the expected number of host–fungus associations under the random host selection hypothesis, and *T*_j_ is the total number of root samples of plant OTU *j*. Therefore, the deviation of the observed frequencies of association from those expected under the random host selection hypothesis (${\bar{D}}_{i,j}$) is shown as follows:

$${\bar{D}}_{i,j}=\frac{p(\pi_{i,j}|y_{i,j},\alpha_{i,j})-E(\pi_{i,j})}{\sqrt{{\sum_{j=1}^{J}\left(p(\pi_{i,j}|y_{i,j},\alpha_{i,j})-E(\pi_{i,j})\right)}^{2}}}.$$

The value of ${\bar{D}}_{i,j}$ measures the degree of apparent host preference of fungal OTU *i* for host plant OTU *j*, and ranges from −1 to 1: ${\bar{D}}_{i,j}$values >0 indicate that the association between fungal OTU *i* and host plant OTU *j* occurs more frequently than expected under the random host selection hypothesis, whereas ${\bar{D}}_{i,j}$ values <0 indicate that the fungal OTU *i* is associated with plant OTU *j* less frequently than that expected by chance. Mean ${\bar{D}}_{i,j}$ values obtained from the posterior distribution represented the expected degree of apparent host preference of fungal OTU *i* for host plant OTU *j* (hereafter,${\hat{D}}_{i,j}$).

The model was fitted using Markov chain Monte Carlo (MCMC) simulation techniques as implemented in the WINBUGS software (Bayesian Inference Using Gibbs Sampling for Windows, [57]). A total of 100,000 iterations using the over-relax option were “thinned” every 20th realization (resulting in 5,000 realizations) to reduce autocorrelations. The first 40,000 iterations were discarded as a “burn-in” to facilitate convergence with the target posterior distribution. Three chains with different initial values were used to facilitate convergence, and MCMC chain convergence was evaluated using the Gelman–Rubin diagnostic statistic [58, 59]. The WinBUGS code is shown in S1 Appendix.

The values of $\hat{D}$ were also used to determine the taxonomic (phylogenetic) rank of host plants at which the examined ascomycetous or ECM basidiomycetous OTUs showed the greatest apparent host preference. The maximum value of $\hat{D}$was used (Chebyshev distance:${\hat{D}}_{\max}=\max\{{\hat{D}}_{i,1},{\hat{D}}_{i,2},...,{\hat{D}}_{i,j}\}$), which represented the most distinct pattern of apparent host preference observed in fungal OTU *i*, as an indicator of the extent to which a fungal OTU *i* showed apparent host preference. The cut-off similarity with the highest ${\hat{D}}_{\max}$ value represented the level of plant genetic similarity—taken here to be roughly proportional to taxonomic rank—at which host association patterns of the fungal OTUs were determined. For example, a ${\hat{D}}_{\max}$ value would be higher at the 99.8% cut-off similarity of host *rbcL* sequences than that at other cut-off similarities if a fungus had an apparent host preference for a single taxon or closely related group of host taxa. In contrast, the value would be higher at the 97% cut-off similarity if a fungus showed an apparent host preference for a more inclusive group of host taxa. We compared ${\hat{D}}_{\max}$ values between the datasets based on different similarity levels of *rbcL* sequences (99.8%, 99%, 98% and 97%) for ascomycetous and ECM basidiomycetous OTUs. To test whether ${\hat{D}}_{\max}$ values were significantly different among these datasets, Wilcoxon signed-rank tests were performed using the ‘pairwise.wilcox.test’ command in R.

### Evaluation of host genetic distance related to fungal community composition

To evaluate the effect of host genetic distance on the root-associated fungal community composition, the correlation between root-associated fungal OTU composition and the genetic distance between host plant OTUs was assessed by Mantel r statistics in the vegan package of R. Prior to the Mantel analysis, the sample-level matrix was divided into two datasets according to the taxonomy of the host OTUs; one dataset contained samples identified as dipterocarp plants (dipterocarp dataset), whereas the second consisted of the samples identified as non-dipterocarp plants (non-dipterocarp dataset). In the Mantel analysis, pairwise fungal community dissimilarity between host plant OTUs was quantified by means of β-diversity for the dipterocarp and non-dipterocarp datasets, separately. Because the α-diversity of fungal OTUs varied among samples, the β -diversity was calculated based on the Raup–Crick metric (‘raupcrick’ command in the vegan package of R), which is less likely to be affected by variance in α-diversity than other metrics of β -diversity [60]. After aligning the *rbcL* sequences using the multiple sequence alignment program MAFFT [61], the genetic distance between the *rbcL* sequences of host plant OTUs was calculated with the most appropriate substitution model using PAUP* 4.0b10 [62], S5 Dataset. One *rbcL* contig (P023), which was largely differentiated from other contigs, was excluded from the analysis. The most appropriate substitution model was identified as the general time reversible substitution model with gamma correction for among-site rate variation and a correction for significant invariable sites (GTR +G+I) by the Akaike Information Criterion (AIC) using jModeltest 2.14 [63]. Significance of the Spearman's rank correlation coefficient between fungal community dissimilarity and host plant genetic distance was tested on the basis of randomizations under a null model that assumed no correlation between two distance variables (9,999 permutations). For each dipterocarp and non-dipterocarp dataset, the β-diversity was also calculated for the subsets of only ascomycetous, basidiomycetous, or ECM basidiomycetous fungi, and an additional Mantel test was conducted on each subset.

Spatial autocorrelation in the distribution of plant and fungal OTUs may be caused by endogenous factors (e.g., conspecific attraction, dispersal limitation) and/or exogenous factors (e.g., autocorrelated environment), and can affect the results of the abovementioned tests. We therefore examined the correlation between pairwise fungal community dissimilarity and geographic distance based on the Mantel test. The spatial autocorrelation of each fungal OTU was also evaluated with the Moran's *I* statistic using the ‘Moran.I’ command in the ape package of R.

In addition, partial Mantel tests were used to evaluate whether the patterns observed in the relationship between fungal community dissimilarity and host genetic distance would hold after controlling for the effects of potential spatial autocorrelation in the dataset. The partial Mantel tests were conducted with 9,999 permutations using the ‘mantel.partial’ command in the vegan package. Similarly, partial Mantel tests were used to assess the relationship between fungal community structure and geographic distance after controlling for the effects of host genetic distance.

## Results

### Clustering and identification of plant and fungal OTUs

In total, 79,558 reads of the chloroplast *rbcL* and 103,528 reads of the ITS2 region (55,355 in the first run and 48,173 in the second run; DDBJ Sequence Read Archive: DRA002296-DRA002304) were obtained from 546 terminal root samples, after removing singletons and low-quality or potentially chimeric sequencing reads. The total number of plant OTUs and fungal OTUs were 158 (99.8% similarity cutoff) and 2067 (95% similarity cutoff), respectively, after excluding singletons. After rarefying the dataset to the depth of 100 reads and removing 104 root samples with less than 100 reads, the total number of plant and fungal OTUs were reduced to 148 and 1757, respectively.

Of the 148 plant OTUs, 90.3% were identified to the family level, S6 Dataset, S1 Fig. Among the potential ECM host lineages in the plot, Dipterocarpaceae were the most common (183/399, or 45.9% of total samples); Fagaceae were not detected in our samples. Members of the Myrtaceae and Fabaceae, which include several ECM lineages, accounted for small percentages of the root samples (5.0% (20/399) and 4.5% (18/399), respectively). Within putative non-ECM host lineages, members of the Burseraceae and Euphobiaceae were relatively frequent, making up 9.8% (39/399) and 8.0% (32/399), respectively, of the root samples.

Of the 1757 fungal OTUs detected, 88.2% were identified at the phylum level, 38.0% at the ordinal level, and 25.7% at the family level, S6 Dataset, S2 Fig. The majority of fungal OTUs belonged to the Dikarya (Ascomycota and Basidiomycota). The Ascomycota were the most diverse phylum, accounting for 79.8% (1245/1561) of fungal OTUs. The Basidiomycota accounted for 18.4% (288/1561) of the community members, of which 44.1% (127/288) were putative ECM Basidiomycota. The most diverse groups within Dikarya were the orders Helotiales (11.5% [77/667]) and Agaricales (9.7% [65/667]), the families Herpotrichiellaceae (12.2% [55/451]) and Russulaceae (11.3% [51/451]), and the genera *Russula* (10.3% [24/234]) and *Chaetosphaeria* (7.3% [17/234]). The Glomeromycota and Chytridiomycota were relatively rare, making up 1.5% (23/1561) and 0.3% (5/1561) of the OTUs, respectively, although it should be noted that rarity of the non-Dikarya taxa was possibly influenced by PCR primer bias.

### Comparison of fungal community composition between dipterocarp and non-dipterocarp plants

The Ascomycota and Basidiomycota were frequently observed in the root samples of most host plants, whereas the Glomeromycota and Chytridiomycota were relatively rare in root samples (Table 1). The Glomeromycota, also known as arbuscular mycorrhizal fungi, were much more frequent in the root samples of non-dipterocarp (12.7%) than in dipterocarp host plants (1.6%). In contrast, putative ECM Basidiomycota OTUs occurred more frequently in the root samples of dipterocarp (85.2%) than in non-dipterocarp host plants (45.2%). At the family level, several putatively ectomycorrhizal fungal taxa, such as Thelephoraceae and Cortinariaceae, occurred more frequently in the root system of dipterocarp (54.1% and 8.2%) than in non-dipterocarp host plants (9.3% and 0.4%). The Russulaceae were common in the root system of both Dipterocarpaceae (46.4%) and the other host plants (32.4%). Ascomycetous OTUs were found in all the root samples of both dipterocarp and non-dipterocarp host plants.

**Table 1:** Frequency of occurrence of each fungal taxon in root samples of dipterocarp (Dip) and non-dipterocarp (non-Dip) host plants.

Phylum	Fungal taxon	Total (%) [n = 442]	Dip (%) [n = 183]	non-Dip (%) [n = 259]
Ascomycota	(in total)	100.0	100.0	100.0
	Herpotrichiellaceae	33.0	34.4	32.0
	Chaetosphaeriaceae	31.0	32.8	29.7
	Hypocreaceae	25.1	28.4	22.8
	Nectriaceae	12.9	10.4	14.7
	Trichocomaceae	9.7	6.0	12.4
	Dermateaceae	8.6	3.8	12.0
	Ophiostomataceae	6.6	8.2	5.4
Basidiomycota	(in total)	82.8	93.4	75.3
	(ECM taxa) ^*^	61.8	85.2	45.2
	Russulaceae	38.2	46.4	32.4
	Thelephoraceae^*^	27.8	54.1	9.3
	Tricholomataceae^*^	14.5	7.7	19.3
	Cantharellaceae	6.6	7.1	6.2
	Cortinariaceae^*^	3.6	8.2	0.4
	Amanitaceae	2.0	2.7	1.5
	Sebacinaceae	1.6	2.7	0.8
	Clavariaceae	1.4	1.1	1.5
Glomeromycota	(in total) ^*^	8.1	1.6	12.7
Chytridiomycota	(in total)	1.4	0.5	1.9

* Frequency of occurrence was significantly different between dipterocarp and the non-dipterocarp plants (Fisher’s exact test, α = 0.05, using a Holm correction).

### Accumulation curve in OTU richness of root-associated fungi

**Fig 1:**
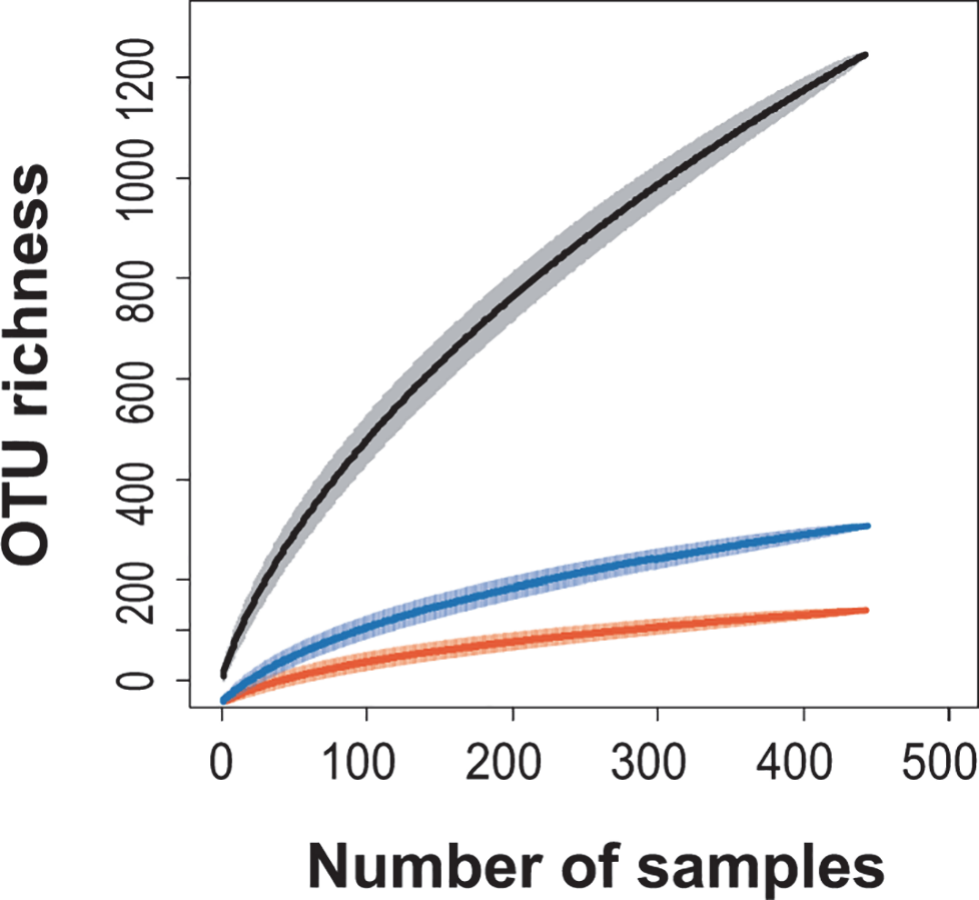
Accumulation curve of operational taxonomic unit (OTU) richness of Ascomycota (black), Basidiomycota (blue), and ectomycorrhizal Basidiomycota taxa (orange) observed in the study site. The shaded areas represent 95% confidence intervals.

The accumulation curve of all fungal taxa, including Ascomycota, Basidiomycota, and ECM Basidiomycota, did not reach an asymptote, indicating that actual OTU richness in the study site was likely greater than observed in the present sampling effort (Fig 1). The graphs also demonstrate that ascomycetous OTUs were accumulating at much higher rates with increasing sampling effort than were the basidiomycetous OTUs.

### Apparent host preference of root-associated fungi

**Fig 2:**
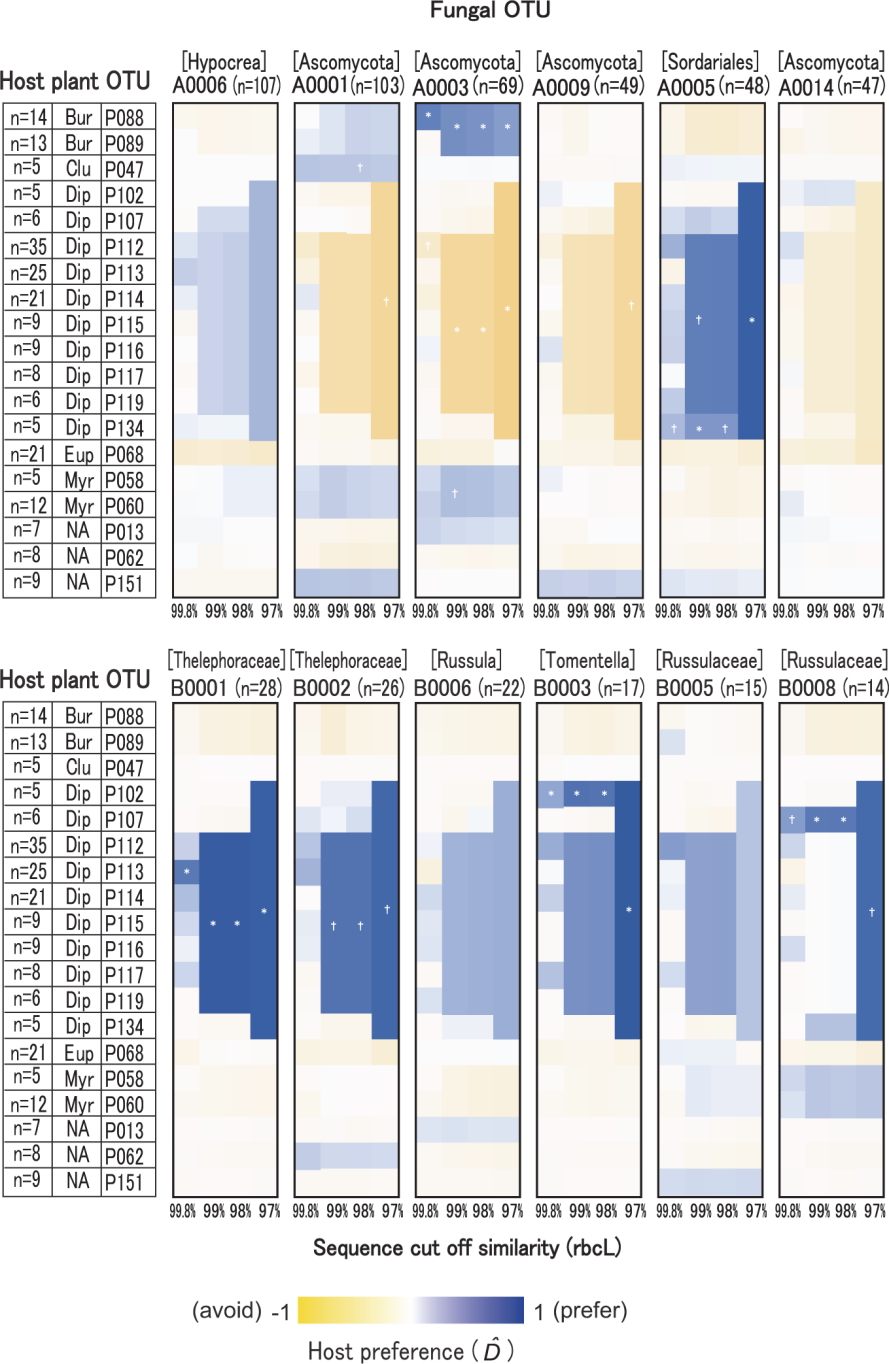
Posterior distribution of $\hat{D}$ values (expected degrees of apparent host preference) for representative root-associated fungal OTUs detected in the root samples. **$\hat{D}$**values are shown separately for the datasets of respective cut-off similarities of host plant sequences. Host plant OTUs represented in ≥5 samples are shown. Color gradients indicate $\hat{D}$ value of each fungal OTU. Plant OTU families are abbreviated as Bur (Burseraceae), Clu (Clusiaceae), Dip (Dipterocarpaceae), Eup (Euphorbiaceae), Myr (Myrtaceae), and NA (family information was not available). An asterisk and a dagger indicate a 99% and 95% credible interval that does not contain 0, respectively.

Multinomial-Dirichlet modeling revealed that many of the examined ascomycetous OTUs had apparent preferences for either dipterocarp or non-dipterocarp species, and with different strengths, depending on OTU. Most basidiomycetous OTUs had apparent preferences for dipterocarp hosts (Fig 2, Table 1, S3 and S4 Figs).

Among the ascomycetous OTUs, A0005 (Sordariales sp.) showed a distinct apparent preference for the OTUs of dipterocarps at all the examined cut-off similarities of *rbcL* sequences, whereas A0003 showed significant apparent preference for OTUs of Burseraceae at all the examined cut-off similarities of *rbcL* sequences (Fig 2, S3 Fig). Patterns similar to A0005 were observed in many ascomycetous OTUs, such as A0004, A0007, A0010, A0015, A0098, A0038 (all Ascomycota sp.) and A0012 (Chaetosphaeriaceae sp.) (Fig 2, S3 Fig). However, these fungi were also detected in the roots of non-dipterocarp plants and the apparent trends in host preferences were not statistically significant. In addition, ascomycetous OTUs A0001 (Ascomycota sp.) and A0031 (Chaetothyriales sp.) showed weak apparent host preference for OTUs of the Clusiaceae and Myrtaceae S3 Fig. Host association patterns of the remaining ascomycetous OTUs were much less obvious, S3 Fig.

Among the basidiomycetous OTUs, the *Tomentella*-*Thelephora* OTUs (B001, B002, B003, and B004) and the *Cortinarius* OTU (B007), all considered to be ECM fungi, had distinct apparent host preferences for OTUs of dipterocarps (Fig 2, S4 Fig). The host association pattern was evident at all the examined *rbcL* sequence similarity levels, but was particularly distinct at the 97%–99% level. Among the *Russula*-*Lactarius* OTUs (also putative ECM taxa), B008 showed a significant apparent preference for dipterocarp OTUs at all the examined cut-off similarities of *rbcL* sequences. Other *Russula*-*Lactarius* OTUs, such as B0006, B0003, B0005, B0022, B0012, B0018, and B0025, had less distinct apparent preferences for the dipterocarp OTUs and their host association patterns were not statistically significant.

**Fig 3:**
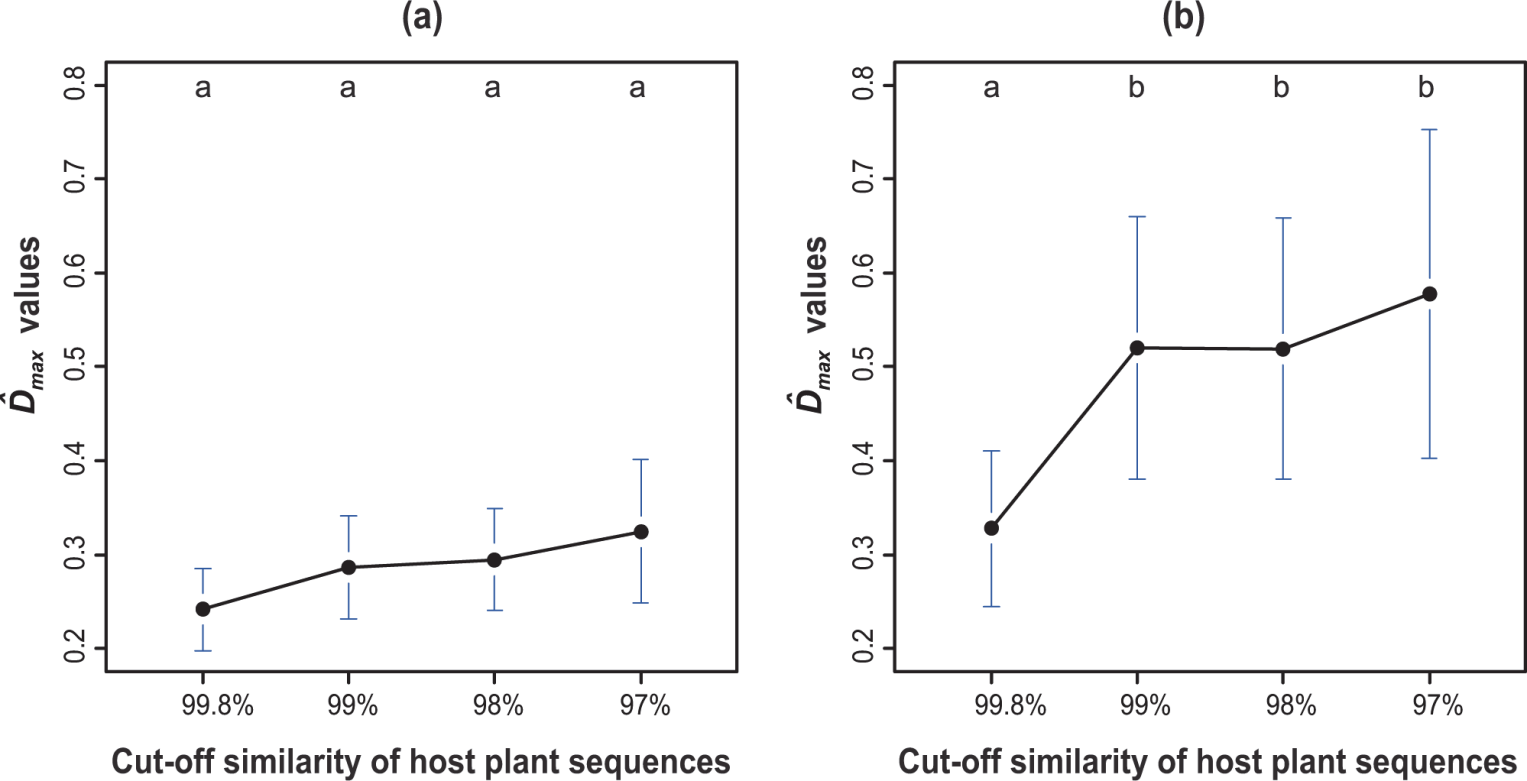
values (index of degrees of apparent host preference at the particular taxonomic rank of host plants) within the respective OTUs of (a) Ascomycota and (b) putative ectomycorrhizal Basidiomycota. ${\hat{D}}_{\max}$ These values were calculated separately for the datasets based on the different cut-off similarities of host plant sequences (97%, 98%, 99%, and 99.8%). The values of $\hat{D}$ correspond to those of Fig 2 and Fig 3, respectively. Different letters indicate a statistically significant difference between cut-off levels of host plant sequences (α = 0.05, using a Holm correction).

The genetic similarity levels of host plants at which the ECM basidiomycetous OTUs exhibited the most distinct host association pattern were much more evident than those for the ascomycetous OTUs. ${\hat{D}}_{\max}$values observed in ascomycetous OTUs were not significantly different between the datasets based on different cut-off similarities of host *rbcL* sequences. However, ${\hat{D}}_{\max}$values observed in basidiomycetous OTUs were significantly lower in the dataset based on a 99.8% cut-off *rbcL* sequence similarity than for other cut-off similarity levels (Fig 3). In addition, values were much lower for ascomycetous OTUs (average values were 0.241, 0.286, 0.295 and 0.325 for 99.8%, 99%, 98% and 97% cut-off levels, respectively) than for the ECM basidiomycetous OTUs (average values were 0.327, 0.520, 0.518, and 0.577 for 99.8%, 99%, 98%, and 97% cut-off levels, respectively).

### Association between host genetic similarity and root-associated fungal community similarity

Standard Mantel tests indicated significant correlations between community dissimilarity of fungi and host genetic distance in several, but not all, combinations of plant taxonomic groups and fungal taxonomic/functional groups (Table 2). Correlations between the dissimilarity in OTU composition of all fungal taxa and host genetic distance were not statistically significant in both the dipterocarp (R = −0.037, P = 0.886) and non-dipterocarp datasets (R = 0.003, P = 0.436). Similarly, there were no significant correlations between dissimilarity in ascomycetous OTU composition and host genetic distance for both the dipterocarp (R = −0.023, P = 0.756) and non-dipterocarp datasets (R = 0.028, P = 0.101). However, community dissimilarity of basidiomycetous OTUs increased significantly with increasing host genetic distance for the dipterocarp dataset (R = 0.062, P = 0.002), but not for the non-dipterocarp dataset (R = 0.021, P = 0.122). Similarly, ECM Basidiomycota demonstrated significant positive correlations between community dissimilarity and host genetic distance for the dipterocarp dataset (R = 0.078, P < 0.001) but not for the non-dipterocarp dataset (R = −0.015, P = 0.876). The remaining basidiomycetous taxa showed no statistically significant correlations between community dissimilarity and host genetic distance for both the dipterocarp (R = -0.010, P = 0.674) and non-dipterocarp dataset (R = 0.031, P = 0.054).

**Table 2:** Results of standard and partial Mantel tests between community dissimilarity of fungi and genetic distance of host plants. Mantel tests were performed for dipterocarp (dip) and non-dipterocarp (non-dip) datasets separately. In partial Mantel tests, correlation between the two variables was tested after controlling for the effect of geographical distance.

Taxa	Dataset	Statistical analysis	Mantel r	P value
All fungal taxa	dip	Standard Mantel	-0.037	0.886
	dip	Partial Mantel	0.004	0.424
	non-dip	Standard Mantel	0.003	0.436
	non-dip	Partial Mantel	0.001	0.485
Ascomycota	dip	Standard Mantel	-0.023	0.756
	dip	Partial Mantel	-0.023	0.757
	non-dip	Standard Mantel	0.028	0.101
	non-dip	Partial Mantel	0.026	0.116
Basidiomycota	dip	Standard Mantel	0.062	0.002
	dip	Partial Mantel	0.063	0.002
	non-dip	Standard Mantel	0.021	0.122
	non-dip	Partial Mantel	0.019	0.140
ECM Basidiomycota	dip	Standard Mantel	0.078	<0.001
	dip	Partial Mantel	0.079	<0.001
	non-dip	Standard Mantel	-0.015	0.820
	non-dip	Partial Mantel	-0.018	0.876
Basidiomycota	dip	Standard Mantel	-0.010	0.674
(excluding ECM taxa)	dip	Partial Mantel	-0.009	0.659
	non-dip	Standard Mantel	0.031	0.054
	non-dip	Partial Mantel	0.030	0.059

Standard Mantel tests also showed significant correlations between community dissimilarity of fungi and geographic distance, except for the dipterocarp dataset with ascomycetous OTUs, S1 Table. Moreover, Moran's *I* statistics indicated that weakly aggregated spatial distributions were observed for about half of the frequently observed fungal OTUs S5 Fig. However, partial Mantel tests, in which the relationship between fungal community dissimilarity and host genetic distance was tested after controlling for the effects of the geographical distance, showed almost the identical results as those of the standard Mantel tests (Table 2).

## Discussion

Our results revealed that tropical trees in the Lambir Hills National Park host a large number of Dikarya OTUs in their root systems. Within the Dikarya OTUs, Ascomycota and Basidiomycota displayed distinct characteristics regarding their biodiversity and host association. We here discuss patterns of the biodiversity and host association of ascomycetous and basidiomycetous OTUs with special emphasis on the difference between root-associated Ascomycota and putative ECM Basidiomycota.

### Biodiversity and host association of root-associated Ascomycota

In the molecular identification, the majority of root-associated Ascomycota in this forest were not assigned to the genus or species rank, S6 Dataset. However, it should be noted that members of the *Cenococcum geophilum* complex, which is one of the most common and well-known ECM ascomycetous taxa, were rarely found in the present study, S3 and S6 Datasets, in contrast to previous work performed in a dry deciduous dipterocarp forest [31].

In this forest system, ascomycetous fungi were found to be species-rich and almost ubiquitous in root systems. These fungi were also characterized by several highly abundant OTUs and a large number of rare OTUs (Fig 1A, S2 Dataset), in agreement with previous studies [42, 43, 64, 65]. The OTU richness described here cannot be directly compared with previous studies because of the different methodologies used for assessing fungal OTU richness (e.g., different molecular markers, PCR primers, cut-off similarity, and/or clustering programs). Reanalyses of the previous studies using the same molecular markers (ITS2 region), cut-off similarity thresholds (95%), and clustering programs (Assams) as done in this work indicated a much lower OTU richness of Ascomycota in a warm temperate forest in Japan and in a cool temperate forest in Japan, S6 Fig. These results confirm the high diversity of root-associated Ascomycota, the majority of which may be represented by root-endophytic Ascomycota, in a tropical dipterocarp rainforest.

Based on Dirichlet-multinomial modeling, we found that most root-associated Ascomycota were characterized by having low or moderate levels of apparent host preference (Fig 2, S3 Fig), in agreement with previous studies [24, 42, 43]. However, results of the standard and partial Mantel tests suggested that ascomycetous OTU composition was not significantly structured by host plant genetic distances (Table 2), and thus host plant genetic distances did not play a crucial role in ascomycetous OTU composition or biodiversity (but it should also be noted that short sequencing reads of the *rbcL* might not exactly reflect the degrees of divergence between host plants). Instead of host genetic distances, ascomycetous communities with non-dipterocarp hosts were structured by geographic proximity, S1 Table. The remarkably high biodiversity of putative root-associated Ascomycota in tropical rainforests might be better explained by other factors such as the edaphic environmental heterogeneity in this forest [18].

### Biodiversity and host association of ECM Basidiomycota

These results showed that the biodiversity of ECM Basidiomycota was much lower than that of root-associated Ascomycota (Fig 1). The OTU richness of ECM Basidiomycota in this forest was similar to, or slightly greater, than those observed in comparable studies conducted in a warm temperate forest [55] and a cool temperate forest [45], S6 Fig. These results suggest that biodiversity of ECM Basidiomycota in tropical rainforests is not very high compared with that in warm- and cool-temperate forests.

Results from the Dirichlet-multinomial modeling indicated that ECM Basidiomycota had apparent host preferences for dipterocarp trees, and their preferences were determined at higher levels of host genetic dissimilarity (e.g., perhaps reflecting genus or family-level, rather than species-level, apparent host preference). Comparison of ${\hat{D}}_{\max}$ (Fig 3) indicated that host association patterns of ECM Basidiomycota were distinct for a range of *rbcL* sequences (97%–99%) representing interspecific to intergeneric differences [66, 67]. These results were generally consistent with those of previous studies, which showed that ECM fungi generally had host preference at the genus- or family- level rather than at the species-level [20,40, 68, 69]: however, note that the present study did not show directly at which host taxonomic levels ECM fungi show host preference.

It was also found that the *Tomentella*-*Thelephora* and *Cortinarius* OTUs showed more distinct patterns in apparent host preferences for closely related taxa within the Dipterocarpaceae than the *Russula*-*Lactarius* OTUs (Fig 2, S4 Fig). Since ECM fungi in the study site were unlikely to form ECM partnerships (i.e., mutualistic relationships) with many different kinds of non-dipterocarp plants, including non-ECM trees, the latter fungal taxa might lack strict host specificity during initial hyphal/root contacts and might have accidental contact with non-ECM plants. However, further anatomical and physiological studies are required to determine whether these respective pairs of fungal and plant OTUs formed mutualistic relationships.

The present study indicated that host phylogenetic diversity could influence the OTU composition and regional biodiversity of ECM Basidiomycota. ECM fungi showed preferences at higher levels of host genetic dissimilarity, and the OTU composition of putative ECM Basidiomycota between dipterocarp taxa became more uniform with decreasing genetic distance between host plants (Table 2). These findings also suggest that the biodiversity of the ECM fungi in dipterocarp rainforests would be increased by host phylogenetic diversity [70].

Our work has implications for the understanding of the relationship between ECM fungal diversity and host phylogenetic diversity. This study showed that host phylogenetic diversity could account for the moderate or relatively low local biodiversity of ECM fungi in a tropical rainforest. However, it should also be noted that communities of root-associated Basidiomycota, including both ECM and non-ECM Basidiomycota, appeared to be structured by geographic proximity, S1 Table, suggesting that community structure of root-associated Basidiomycota in this forest is not only influenced by host specificity but also by other factors, such as edaphic or microclimatic factors. Moreover, we acknowledge that the results for the Lambir Hills National Park are not necessarily indicative of the global biodiversity pattern of ECM fungi, and that further comparative studies based on identical pyrosequencing methods are needed.

### Biodiversity and distribution of non-Dikarya taxa

Although the present study targeted Dikarya taxa, 28 non-Dikarya OTUs, including 23 OTUs from the Glomeromycota (arbuscular mycorrhizal fungi), were detected in samples. This may be due to closer matches between Dikarya ITS sequences and the PCR primers used in this study, or possibly to the relatively higher DNA amounts of Dikarya taxa in the root systems. Nevertheless, further investigations that use specific primers for Glomeromycota are required to determine the biodiversity and ubiquity of these fungi in a dipterocarp rainforest.

## Conclusion

This study demonstrated that trees in the paleotropical lowland rainforests of Southeast Asia harbored extremely diverse root-associated Ascomycota that were characterized by low to moderate level of apparent host preference. ECM Basidiomycota showed less diversity and greater levels of host preference, and their associations with host dipterocarp trees correlated with host genetic distance. This study also implied that not only host specificity but also other factors operating at small ecological scales (e.g., edaphic or microclimatic factors) could account for community structure of root-associated fungi in this forest. The present findings suggested that tropical rainforests are likely biodiversity hotspots for root endophytes or other root-associated Ascomycota. In addition, host phylogenetic diversity appears to be one of the most critical issues for understanding the global pattern of ECM fungal biodiversity, as previously suggested by Tedersoo and colleagues [69].

## Supporting Information

S1 Appendix WinBUGS code used to evaluate apparent host preference of representative operational taxonomic units (OTUs) of root-associated fungi. (DOCX)

S1 Dataset Consensus sequences of plant and fungal operational taxonomic units (OTUs) in Fasta format. An initial letter of each OTU name represents the taxonomic classification (P: Plant, A, Ascomycota: B, Basidiomycota; C, Chytridiomycota; G, Glomeromycota; N, phylum unknown).
(FASTA)

S2 Dataset Data matrix that depicts the total sequence reads of respective fungal operational taxonomic units (OTUs) in each sample (a) before or (b) after the dataset was rarefied to a depth of 100 reads. (XLSX)

S3 Dataset A binary data matrix that depicts the presence/absence of respective fungal operational taxonomic units (OTUs) after the dataset of sequence reads was rarefied to a depth of 100 reads. (XLSX)

S4 Dataset Data matrix that represents associations between plant and fungal operational taxonomic units (OTUs), in which plant OTUs were clustered with similarity cut-offs of (a) 99.8%, (b) 99%, (c) 98% and (d) 97%. In the matrix, rows represent fungal OTUs and columns represent plant OTUs.
(XLSX)

S5 Dataset Genetic distances of chloroplast *rbcL* sequences between host plant operational taxonomic units (OTUs). (XLSX)

S6 Dataset Results of taxonomic assignment of (a) host plant and (b) fungal operational taxonomic units (OTUs) using the Claident program. (XLSX)

S1 Fig Proportional distribution of different families in host plant operational taxonomic units (OTUs). (EPS)

S2 Fig Proportional distribution of different taxonomic groups in fungal operational taxonomic units (OTUs). (EPS)

S3 FigPosterior distribution of $\hat{D}$ values (expected degrees of host association) for the most common ascomycetous OTUs detected in the root samples.
$\hat{D}$values are shown separately for the datasets of respective cut-off similarities of host plant sequences. Host plant OTUs with samples of ≥5 are shown. Color gradients indicate $\hat{D}$ value of each fungal OTU. The families of each host plant OTU are abbreviated as Bur (Burseraceae), Clu (Clusiaceae), Dip (Dipterocarpaceae), Eup (Euphorbiaceae), Myr (Myrtaceae), and NA (family information was not available). An asterisk and a dagger indicate 99% and 95% credible intervals that do not contain 0, respectively.(EPS)

S4 FigPosterior distribution of $\hat{D}$ values (expected degrees of host association) for the most common OTUs of putative ectomycorrhizal Basidiomycota detected in the root samples.
$\hat{D}$values are shown separately for the datasets of respective cut-off similarities of host plant sequences. Host plant OTUs with samples of ≥5 are shown. Color gradients indicate $\hat{D}$ value of each fungal OTU. The families of each host plant OTU are abbreviated as Bur (Burseraceae), Clu (Clusiaceae), Dip (Dipterocarpaceae), Eup (Euphorbiaceae), Myr (Myrtaceae), and NA (family information was not available). An asterisk and a dagger indicate 99% and 95% credible intervals that do not contain 0, respectively.(EPS)

S5 Fig Spatial distribution of representative operational taxonomic units (OTUs) of root-associated fungi at a 600 m line transect of the study site. Depth of color indicates the frequency of occurrence of each fungal OTU at different distance classes.
(EPS)

S6 Fig Accumulation curve of operational taxonomic unit (OTU) richness of (a) Ascomycota, (b) Basidiomycota, and (c) ectomycorrhizal Basidiomycota taxa observed in a tropical dipterocarp forest (black; this study), a warm temperate forest (blue; 35°02'N; data from previous work [52]), and a cool temperate forest (orange; 42°40'N; data from previous work [42]). The shaded areas represent 95% confidence intervals.
(EPS)

S1 Table Results of standard and partial Mantel tests between community dissimilarity of fungi and geographic distance. Mantel tests were performed for dipterocarp (dip) datasets and non-dipterocarp (non-dip) datasets, separately. In partial Mantel tests, correlation between the two variables was tested after controlling for the effect of genetic distance of host plants.
(DOCX)
