# Supplementary material for: Contrasting Diversity and Host Association of Ectomycorrhizal Basidiomycetes versus Root-Associated Ascomycetes in a Dipterocarp Rainforest
Source: PLoS One. 2015 Apr 17;10(4):e0125550. doi: 10.1371/journal.pone.0125550 (PMC4401655; doi:10.1371/journal.pone.0125550)
Supplement: S1 Appendix — (DOCX) [file pone.0125550.s001.docx]

# Model for assessing host preference of root-associated fungi based on plant × fungal matrix in which plant OTUs were clustered with similarity cut-off of 99.8% (Data S4a)

model

{

for (i in 1:36)

{

pi[i,1:148]~ ddirch(alpha[i,]) # Dirichlet distribution

Y[i,1:148]~ dmulti(pi[i,],N[i]) # Multinomial distribution

D[i]<- sqrt(sum(d2[i,])) # Euclidean distance between empirical data vector and hypothetical data vector

for (j in 1:148)

{

alpha[i,j]<- T[j]*N[i]/442 # The expected number of host-fungus association under the random host selection hypothesis

d[i,j]<- pi[i,j]-alpha[i,j]/sum(alpha[i,])

d2[i,j]<- d[i,j]*d[i,j]

Dbar[i,j]<- d[i,j]/D[i] # Index of host preference

}

}

}

ist(E=c(3,2,1,1,1,3,1,1,1,1,2,7,2,1,1,1,1,1,1,2,1,1,3,1,1,2,2,1,1,1,2,1,1,1,3,1,3,1,1,1,4,1,4,1,5,3,2,1,3,2,1,1,1,1,1,5,2,12,3,8,2,3,1,21,1,1,1,1,1,2,1,2,1,1,2,1,4,3,2,1,2,1,1,14,13,4,2,2,1,1,1,1,3,3,1,1,5,1,2,1,4,6,4,2,1,35,25,21,9,9,8,3,6,4,2,2,2,1,1,1,1,1,1,1,1,5,3,2,1,1,1,1,3,2,1,1,4,4,1,1,1,9,2,1,1,4,3,3),

N=c(115,112,107,103,89,85,77,69,60,56,56,52,49,48,48,47,47,46,40,39,37,37,34,31,28,26,22,17,15,14,13,13,12,11,11,11),

Y=structure(.Data=c(1,1,0,0,0,0,1,1,0,0,0,1,1,0,0,0,1,1,0,0,1,1,0,0,0,0,0,0,0,0,0,1,0,0,2,0,0,0,0,0,2,0,1,0,2,1,1,0,2,0,1,0,1,0,0,1,0,4,2,3,0,0,1,2,0,0,1,0,1,2,0,1,0,0,0,1,1,0,0,0,0,0,1,0,2,0,1,1,0,0,0,1,2,0,0,0,2,0,1,0,1,1,2,0,0,12,12,4,3,2,2,2,1,1,1,1,0,0,0,1,0,0,0,0,0,0,0,2,1,0,1,1,2,1,0,1,0,2,0,0,0,1,0,0,0,1,0,1,

1,0,1,0,0,1,0,1,0,0,0,2,0,0,0,1,0,0,0,1,0,0,1,0,1,1,1,0,0,0,0,1,1,0,0,0,2,1,0,0,0,0,1,0,0,0,0,0,0,0,0,0,0,0,0,4,0,4,1,3,0,0,1,1,0,0,0,0,0,0,0,0,0,0,0,0,0,1,0,0,1,0,0,0,4,0,1,0,1,0,1,0,1,0,0,0,1,0,0,1,0,3,1,1,1,13,10,7,2,2,2,1,2,1,0,1,0,1,1,0,1,0,0,0,0,2,1,0,1,0,1,0,3,0,0,0,0,2,0,0,0,4,0,1,0,1,1,1,

0,0,1,0,0,1,0,0,0,0,0,2,0,0,0,1,0,1,0,1,0,0,1,0,1,0,0,0,0,0,1,1,0,1,0,1,0,0,0,0,2,0,0,0,1,2,1,1,1,0,0,0,0,0,0,1,1,3,1,2,0,0,1,1,1,0,0,0,1,0,0,1,0,0,0,1,2,2,0,1,1,0,0,2,3,1,0,0,1,0,1,0,0,1,0,0,1,0,0,1,1,1,2,1,1,10,9,6,1,2,1,1,1,2,0,0,1,0,0,1,0,0,0,0,0,2,0,1,0,0,0,0,2,0,0,0,1,0,1,1,0,1,0,0,0,2,1,2,

0,0,0,0,0,2,0,0,0,0,1,0,0,1,1,0,0,0,0,1,0,0,2,0,0,0,1,0,1,0,0,0,1,0,0,1,1,1,1,0,0,1,2,0,5,0,0,0,2,0,0,0,0,1,1,3,0,5,0,0,0,0,0,4,0,0,0,0,0,0,0,0,1,0,0,0,1,1,2,1,0,0,0,3,4,2,0,0,1,1,0,1,1,0,1,0,0,0,0,0,0,1,1,1,0,3,3,6,1,2,0,0,1,0,0,1,1,0,0,0,1,1,0,0,1,0,0,1,0,1,0,1,0,0,0,0,2,2,0,0,0,6,1,0,1,3,1,1,

1,0,0,0,0,1,0,1,0,0,0,1,0,1,0,0,1,0,0,0,0,0,0,0,0,0,0,0,0,0,0,1,0,0,0,0,0,0,0,0,2,0,1,0,0,1,1,1,0,1,0,0,0,0,0,0,0,4,0,2,0,1,1,4,0,0,0,0,0,0,0,0,0,0,0,1,1,2,0,0,0,1,0,2,4,0,0,0,0,0,1,0,0,0,0,0,0,1,0,1,2,3,0,0,1,10,7,5,1,2,1,0,1,1,0,0,0,0,0,1,1,0,1,0,0,2,1,1,0,0,0,1,1,0,0,0,2,0,0,0,0,2,0,0,0,0,0,2,

1,0,0,0,0,2,0,0,0,0,2,0,0,1,1,0,0,0,1,0,1,0,0,0,0,0,0,0,1,1,0,0,0,0,0,0,1,0,0,0,1,1,1,0,3,0,0,0,2,2,1,1,0,1,0,1,2,2,1,6,0,0,0,2,0,0,0,1,0,0,0,0,0,0,0,0,0,1,0,0,0,0,0,1,3,0,0,0,0,0,0,0,0,0,0,0,0,0,0,0,1,2,2,0,0,7,4,5,4,1,3,1,1,0,0,0,0,0,0,0,1,0,1,0,0,1,0,0,0,0,0,0,0,0,0,0,1,0,1,0,0,2,0,0,0,0,2,0,

2,0,0,1,0,0,0,0,0,0,0,1,0,0,0,0,0,0,0,0,0,0,0,0,0,0,1,0,0,0,0,1,0,1,1,1,0,0,0,0,1,1,0,0,0,1,0,0,0,0,0,0,1,0,0,1,0,3,1,1,1,1,0,0,0,0,1,0,1,1,0,1,0,1,0,1,2,1,0,0,0,0,0,0,2,0,0,0,0,0,0,0,0,1,0,0,0,1,0,0,1,0,1,1,0,5,9,3,2,3,1,0,1,0,2,0,0,0,0,0,1,0,0,0,0,0,0,0,0,0,0,1,0,1,1,0,1,0,0,0,0,4,0,1,1,1,1,1,

1,1,0,0,0,0,1,0,0,0,0,4,0,0,0,0,0,0,0,1,0,0,0,0,1,2,1,0,0,0,0,0,0,1,2,0,2,1,1,0,0,0,2,1,1,1,0,0,0,0,0,0,0,0,0,3,0,5,1,0,0,0,0,0,0,0,0,0,0,1,0,1,0,0,1,0,1,0,1,0,0,1,1,14,1,0,2,1,0,0,0,0,0,0,0,0,0,0,0,0,0,0,0,0,0,0,2,0,0,2,0,0,0,0,0,0,0,0,0,0,0,0,0,1,0,0,0,0,0,0,0,0,0,1,0,0,1,1,1,0,1,1,0,0,0,0,0,0,

0,0,0,0,0,0,0,1,0,0,0,1,2,0,0,1,0,0,0,0,0,0,0,0,0,0,0,1,0,0,0,1,0,0,1,0,1,0,0,0,0,0,0,0,0,0,0,0,0,0,0,0,0,0,0,3,0,2,0,0,0,0,0,4,0,0,0,0,0,0,0,0,0,0,0,0,0,1,0,0,0,0,0,0,3,1,0,0,0,0,0,0,1,0,0,0,2,0,0,0,0,1,0,0,1,8,4,0,1,2,0,0,0,0,1,0,1,0,0,0,0,0,1,0,0,0,1,1,0,1,1,0,0,0,0,0,1,2,0,0,0,1,0,0,0,2,1,3,

0,0,0,0,0,1,0,0,0,0,0,0,0,1,0,0,0,0,0,0,1,0,0,0,0,0,0,0,1,0,0,0,0,0,0,0,1,0,0,0,1,0,1,0,1,0,0,0,1,2,0,0,0,0,0,0,1,0,0,5,0,0,0,0,0,0,0,1,0,0,1,0,0,0,0,0,0,1,0,0,0,0,0,2,1,0,0,0,0,0,0,0,0,0,0,0,0,0,0,0,1,0,1,0,0,5,2,5,3,1,3,1,0,0,0,0,0,0,0,0,1,0,1,0,0,2,0,0,0,0,0,0,0,0,0,0,1,2,1,0,0,1,0,0,0,0,2,0,

1,0,0,0,0,0,0,0,0,0,1,2,0,0,0,0,0,0,0,0,0,0,0,1,1,0,0,0,0,0,2,1,0,0,1,0,1,0,0,0,1,0,1,0,0,1,0,0,0,0,1,0,1,0,0,0,0,0,1,3,1,1,0,2,0,0,0,0,0,0,0,0,0,0,0,0,0,1,0,0,0,0,1,0,0,0,0,0,0,0,0,0,0,0,0,0,0,0,0,0,1,1,1,0,0,4,7,4,1,1,1,1,1,1,0,0,0,0,0,0,1,0,0,0,1,0,0,0,0,0,0,0,0,1,0,0,0,0,0,0,0,2,0,0,0,1,0,0,

0,1,0,1,0,2,1,0,0,0,0,1,0,0,0,0,0,0,0,0,0,0,1,0,1,0,0,0,0,0,0,1,0,0,0,0,0,0,0,0,2,0,2,0,0,0,0,0,0,0,0,0,0,0,0,0,0,4,1,2,0,0,0,1,0,0,0,0,0,0,0,0,0,0,0,0,0,1,0,0,0,0,0,0,2,0,1,0,1,0,1,0,0,0,0,0,2,0,0,0,0,0,0,0,1,6,1,4,1,1,0,0,1,1,0,0,0,0,1,0,0,0,1,0,0,0,0,1,0,0,0,0,0,0,0,1,0,0,0,0,0,2,0,1,0,0,0,0,

0,0,1,0,0,1,0,0,0,0,1,0,1,0,1,0,0,0,0,1,0,0,1,0,0,0,1,0,1,0,0,0,1,0,0,1,0,1,1,0,0,1,1,0,0,0,0,0,0,0,0,0,0,0,1,1,0,1,0,0,0,0,0,1,0,0,0,0,0,0,0,0,0,0,0,0,0,0,1,1,1,0,0,1,1,1,0,0,0,0,0,1,1,0,0,0,1,0,0,0,0,0,0,0,0,2,2,2,0,2,0,0,0,0,0,0,1,0,0,0,0,0,0,0,0,0,0,0,0,0,0,1,0,0,0,0,1,1,0,0,0,3,1,0,0,4,0,1,

1,0,0,0,0,0,0,0,0,0,0,0,0,0,0,0,1,0,0,0,0,0,1,0,0,0,0,0,0,0,1,0,0,0,0,0,0,0,0,0,0,0,0,0,1,0,0,0,0,0,0,0,0,0,0,0,0,0,0,0,0,0,0,0,0,0,0,0,0,0,0,0,0,0,0,0,0,0,1,0,0,0,0,0,0,0,0,0,0,0,0,0,0,0,0,0,0,0,0,0,0,2,1,1,0,8,1,4,3,3,1,1,3,0,1,0,0,0,0,0,0,0,0,0,0,4,2,1,1,0,0,0,0,1,0,0,0,0,0,0,0,2,0,0,1,0,0,1,

0,0,0,0,0,1,0,0,0,0,0,1,0,0,1,0,0,0,0,1,0,0,0,0,0,0,1,0,0,0,0,0,0,0,0,0,1,0,0,1,0,0,1,1,0,0,0,0,0,0,0,0,0,0,1,1,1,2,0,0,0,1,0,2,0,0,1,0,0,0,0,1,0,0,0,1,1,0,0,0,1,0,0,0,1,1,0,0,0,1,0,0,0,0,0,0,0,0,0,0,0,1,1,0,0,3,4,2,1,1,1,0,1,1,0,0,0,0,0,0,0,0,0,0,1,0,0,0,0,0,0,0,0,0,0,0,0,2,0,0,0,3,0,0,0,1,0,0,

0,0,0,1,0,0,0,0,0,0,0,1,0,0,0,0,0,0,0,0,1,0,0,0,0,0,0,1,1,0,0,1,0,0,2,0,0,0,0,0,1,0,0,0,0,3,1,1,0,0,0,0,0,0,0,0,0,2,0,1,0,0,0,0,0,0,0,0,0,0,0,0,0,0,0,0,1,2,1,0,0,1,1,1,0,0,1,1,0,0,0,0,0,2,0,0,1,0,0,1,0,0,0,0,0,5,3,1,0,0,1,0,0,0,0,0,0,0,0,1,0,0,0,0,0,0,0,0,0,0,0,0,2,0,1,1,0,1,0,0,0,1,0,0,0,0,0,0,

1,0,0,0,0,0,0,1,0,0,0,1,0,0,0,1,0,0,0,1,0,1,0,0,0,0,0,0,0,0,0,0,1,0,0,0,0,1,0,0,0,0,1,1,1,0,0,0,0,0,0,0,0,0,0,0,0,3,0,0,0,0,0,1,0,0,0,0,0,0,0,0,0,0,0,1,0,0,0,0,1,0,0,0,1,0,0,0,0,1,1,0,0,0,0,0,1,0,0,0,1,1,1,0,0,2,6,4,0,0,1,0,1,0,1,0,1,1,0,0,1,0,0,0,0,1,0,0,0,1,0,0,0,0,0,0,1,0,0,0,0,0,0,1,0,1,0,0,

0,0,0,0,0,0,1,0,1,0,0,0,0,1,0,0,0,0,1,0,0,0,0,0,1,0,0,0,1,0,0,1,0,0,0,0,1,0,0,0,1,0,1,0,1,2,0,1,0,0,0,0,1,0,0,0,0,1,1,1,1,2,1,1,0,0,0,0,0,0,0,0,0,0,0,0,0,1,0,0,0,0,1,2,3,0,0,0,0,0,0,1,0,0,0,0,0,0,0,0,0,1,0,0,0,0,3,2,0,0,1,0,0,0,0,0,0,0,0,0,0,0,0,0,0,0,0,0,0,0,0,1,1,0,1,1,0,1,0,0,0,2,0,0,1,0,0,0,

0,0,0,0,0,1,0,0,0,0,0,2,0,0,0,0,0,0,0,0,0,0,0,0,0,0,0,0,0,0,0,0,0,0,0,1,0,0,0,0,0,0,0,0,0,0,0,0,0,0,0,0,0,0,1,0,0,3,0,0,1,1,0,1,0,0,0,0,0,0,0,0,0,0,0,0,0,1,1,0,0,0,1,0,1,0,0,1,0,0,0,1,0,0,0,0,0,0,0,0,2,1,0,0,0,4,3,4,0,2,0,1,1,0,0,0,0,0,1,0,1,0,0,0,0,0,0,0,0,0,0,0,0,0,0,0,0,0,0,0,0,1,0,0,0,1,0,1,

0,0,0,0,0,1,0,1,0,0,0,1,0,0,0,0,0,0,0,0,0,0,0,1,0,0,0,0,1,0,1,0,0,0,1,0,0,0,0,0,0,0,1,0,0,0,0,0,1,0,0,0,0,0,0,1,0,1,1,2,0,0,0,0,0,0,0,0,0,0,0,0,1,0,0,0,1,0,0,0,0,0,0,2,2,0,0,0,0,1,0,0,0,0,0,0,0,0,0,0,0,0,0,0,0,2,3,1,0,1,1,0,1,1,0,0,0,0,0,0,0,0,0,0,0,0,1,0,0,0,0,0,0,0,0,0,0,2,0,1,0,1,0,1,1,1,0,0,

0,0,0,0,0,0,1,1,0,0,1,2,0,0,0,0,0,0,0,0,0,0,0,0,0,1,0,0,0,0,0,0,0,0,2,1,0,0,0,0,0,0,0,0,0,0,0,0,0,0,0,0,0,0,0,1,1,1,0,1,1,0,0,0,0,0,0,0,0,0,0,1,0,0,0,0,0,0,0,0,0,1,0,3,4,0,0,0,0,1,0,0,0,0,0,0,0,0,0,0,0,0,0,0,0,0,2,0,0,0,1,0,1,0,0,0,1,0,0,0,0,0,0,0,0,1,0,0,0,0,0,0,2,0,0,0,1,0,0,1,0,0,0,1,1,1,0,0,

0,0,0,0,0,1,0,0,0,0,0,1,1,0,1,0,0,0,0,0,0,0,0,1,1,0,1,1,0,1,0,0,0,0,0,0,0,0,0,1,0,0,0,0,1,0,0,0,0,0,0,0,0,0,0,0,0,1,0,1,1,0,0,4,0,0,0,0,0,0,0,0,1,1,0,0,1,0,0,0,0,0,0,0,4,0,0,0,0,0,0,0,0,2,0,0,0,0,0,0,0,0,0,0,0,1,4,0,2,0,0,1,0,1,0,0,0,0,0,0,0,0,0,0,0,0,0,0,0,0,0,0,0,0,0,0,0,0,0,0,0,0,0,0,0,0,0,1,

0,0,0,0,0,1,0,0,1,0,1,1,0,0,0,0,0,0,0,0,0,0,0,1,0,0,1,0,0,0,0,0,0,1,0,1,0,0,0,0,1,0,0,0,0,0,0,0,0,0,0,0,0,0,0,0,0,5,0,0,0,0,0,1,0,0,0,0,0,0,0,0,0,1,0,0,0,0,0,0,0,0,0,0,2,0,0,0,0,0,0,0,0,1,0,1,1,0,0,0,0,1,0,0,0,2,3,1,0,1,1,0,0,0,0,0,0,0,1,0,1,0,1,0,0,0,0,0,0,0,0,0,0,0,0,0,0,0,0,0,0,1,0,0,0,0,0,0,

0,0,0,0,0,0,0,0,0,0,0,0,0,0,0,0,0,0,0,0,0,0,0,0,1,0,0,0,0,0,0,0,0,0,0,0,1,0,0,0,0,0,0,0,0,3,1,1,0,0,0,0,0,0,0,1,0,2,1,1,0,0,1,0,0,0,0,0,1,1,1,0,0,0,0,0,0,0,0,0,0,0,0,0,2,0,0,1,0,0,0,0,0,0,0,0,0,0,1,0,1,0,0,0,0,2,2,0,1,0,0,0,0,0,0,0,0,0,0,1,0,0,0,0,0,0,0,0,0,0,0,0,0,1,1,1,0,0,0,0,0,1,0,0,0,0,0,0,

0,0,0,0,0,0,0,0,0,0,0,0,0,0,0,0,0,1,0,0,0,0,0,0,0,0,0,0,0,0,0,0,0,0,0,0,0,0,0,0,0,0,0,0,0,0,0,0,0,0,0,0,0,0,0,0,0,0,0,0,0,0,0,0,1,0,0,0,1,0,0,0,0,0,0,0,0,1,0,0,0,0,0,0,0,0,0,0,0,0,0,0,0,0,0,0,0,0,0,0,0,0,0,0,0,4,9,4,2,1,2,0,0,0,0,0,0,0,0,0,0,0,0,1,0,0,0,0,0,0,0,0,0,0,0,0,0,0,0,0,0,0,0,0,0,1,0,0,

0,0,0,0,0,0,0,0,0,0,0,0,0,0,0,0,0,0,0,0,0,1,0,0,0,0,0,0,0,0,0,1,0,0,0,0,1,0,0,0,0,0,0,0,0,0,0,0,0,0,0,1,0,0,0,0,0,0,1,2,0,0,0,0,0,0,0,0,0,0,0,0,0,0,0,0,0,0,0,0,0,0,0,0,0,0,0,0,0,0,1,0,0,0,0,0,0,0,1,0,1,1,0,0,0,4,4,1,1,1,0,1,0,0,1,0,0,0,0,1,1,0,0,0,0,0,0,0,0,0,0,0,0,0,0,0,0,0,0,0,0,0,0,0,0,0,0,0,

0,0,0,0,0,0,0,0,0,1,0,1,0,0,0,0,0,0,0,0,0,0,0,0,0,0,0,0,0,0,0,0,0,0,0,0,0,0,0,0,2,0,1,0,0,1,0,0,0,0,0,0,0,0,1,0,0,0,0,0,0,0,0,1,0,0,0,0,0,0,0,0,0,0,0,0,0,0,0,0,0,0,0,0,0,0,0,1,0,0,0,0,0,0,0,0,0,0,0,0,1,0,0,0,0,2,0,2,1,1,0,0,1,0,0,1,1,0,0,0,0,0,0,0,0,0,1,0,0,0,0,0,0,1,0,0,0,0,0,0,0,0,0,0,0,1,0,0,

0,0,0,0,0,0,0,0,0,0,0,0,0,0,0,0,0,0,0,0,0,0,0,0,0,0,0,0,0,0,0,0,0,0,0,0,0,0,0,0,0,0,0,0,0,0,0,0,0,0,0,0,0,0,0,0,0,0,0,0,0,0,0,0,0,0,0,0,0,0,0,0,0,0,0,0,0,0,0,0,0,0,0,0,0,0,0,0,0,0,0,0,0,0,0,0,3,1,2,1,0,0,0,0,0,4,1,2,0,0,2,0,0,0,0,0,0,0,1,0,0,0,0,0,0,0,0,0,0,0,0,0,0,0,0,0,0,0,0,0,0,0,0,0,0,0,0,0,

0,0,0,0,0,0,0,0,0,0,0,0,0,0,0,0,0,0,0,0,0,0,0,0,1,0,0,0,0,0,0,0,0,0,0,0,0,0,0,0,0,0,0,0,0,0,0,0,0,0,0,0,0,0,0,0,0,0,1,0,0,0,0,1,0,0,0,0,0,0,0,0,0,0,0,0,0,0,0,0,0,0,0,0,1,0,0,0,0,0,0,0,0,0,0,0,0,0,0,0,0,0,0,0,0,4,1,1,0,0,1,0,0,0,0,0,0,0,0,0,0,0,0,0,0,0,0,0,0,0,0,0,1,0,0,0,0,0,1,0,0,1,0,1,0,0,0,0,

0,0,0,0,0,0,0,0,0,0,0,0,0,0,0,0,0,0,0,0,0,0,0,0,0,0,0,0,0,0,0,0,0,0,0,0,0,0,0,0,0,0,0,0,0,0,0,0,0,0,0,0,0,0,0,1,0,1,0,0,0,0,0,0,0,0,0,0,0,0,0,0,0,0,0,0,0,0,0,0,0,0,0,0,0,0,0,0,0,0,0,0,0,0,0,0,0,0,0,0,0,3,1,1,0,2,0,1,0,1,0,0,0,0,0,0,0,0,0,0,0,0,0,0,0,0,1,1,0,0,0,0,0,0,1,0,0,0,0,0,0,0,0,0,0,0,0,0,

0,0,0,0,0,0,0,0,0,0,0,0,0,0,0,0,0,0,0,0,0,0,0,0,0,0,0,0,0,0,0,0,0,0,0,0,0,0,0,0,0,0,0,0,0,0,0,0,0,0,0,0,0,0,0,0,0,1,0,0,0,0,0,0,0,0,0,0,0,0,0,0,0,0,0,0,0,0,0,0,0,0,0,0,0,0,0,0,0,0,0,0,0,0,0,0,0,0,0,0,0,0,0,0,0,2,2,3,0,1,2,0,0,0,0,1,0,0,0,0,0,0,0,0,0,0,0,0,0,0,0,0,0,1,0,0,0,0,0,0,0,0,0,0,0,0,0,0,

0,0,0,0,0,0,0,0,0,0,0,0,0,0,0,0,0,0,0,0,0,0,0,0,0,0,0,0,0,0,0,0,0,0,0,0,0,0,0,0,0,0,0,0,0,0,0,0,0,0,0,0,0,0,0,0,0,0,0,1,0,0,0,2,0,0,0,0,0,0,0,0,0,0,0,0,0,1,0,0,0,0,0,0,1,0,0,0,0,0,0,0,1,0,0,0,0,0,0,0,0,0,0,0,0,1,1,0,0,0,1,0,1,1,0,0,0,0,0,0,0,0,0,0,0,0,0,0,0,0,0,0,0,0,0,1,0,0,0,0,0,1,0,0,0,0,0,0,

0,0,0,0,0,0,0,0,0,0,0,0,0,0,0,0,1,0,0,0,0,0,0,0,0,0,0,0,0,0,0,0,0,0,0,0,0,0,0,0,0,0,1,0,0,0,0,0,0,0,0,0,0,0,0,0,0,0,0,0,0,0,0,0,0,0,0,0,0,0,0,0,0,0,0,0,0,0,0,0,0,0,0,0,0,0,0,0,0,0,0,0,0,0,0,0,0,0,0,0,1,0,1,0,0,0,2,1,0,1,0,0,1,0,1,0,0,0,0,0,0,0,0,0,0,0,0,1,1,0,0,0,0,0,0,0,0,0,0,0,0,0,0,0,0,0,0,0,

0,0,0,0,0,0,0,0,0,0,0,0,0,0,0,0,0,0,0,0,1,0,0,0,0,0,0,0,0,0,0,0,0,0,1,0,0,0,0,0,0,0,0,0,0,0,0,0,1,0,0,0,0,0,0,0,0,0,0,0,0,0,0,0,0,0,0,0,0,1,0,0,0,0,0,0,0,0,0,0,0,0,0,0,0,0,0,0,0,0,0,0,0,0,0,0,0,0,0,0,0,0,0,0,0,1,2,0,1,0,1,0,0,0,0,0,0,0,0,0,0,0,0,0,0,0,0,1,0,0,0,0,0,0,0,0,0,1,0,0,0,0,0,0,0,0,0,0,

0,0,0,0,0,0,0,0,0,0,0,0,0,0,0,1,0,0,0,0,0,0,0,0,0,0,0,0,0,0,0,0,0,0,0,0,0,0,0,0,0,1,0,0,0,0,0,0,0,0,0,0,0,0,0,0,0,0,0,1,0,0,0,0,0,0,0,0,0,0,0,0,0,0,0,0,0,0,0,0,0,0,0,0,1,0,0,0,0,0,0,0,0,0,0,0,1,0,0,0,0,0,0,0,0,1,2,1,0,0,1,0,0,0,0,0,0,0,0,0,0,0,0,0,0,0,0,0,0,0,0,0,0,0,0,0,1,0,0,0,0,0,0,0,0,0,0,0,

0,0,0,0,0,0,0,0,0,0,0,0,0,0,0,0,0,0,0,0,0,0,0,0,0,0,0,0,0,0,0,0,0,0,0,0,0,0,0,0,0,0,0,0,0,0,0,0,0,0,0,0,0,0,0,0,0,0,0,0,0,0,0,1,0,0,0,0,0,0,0,0,0,0,0,0,0,0,0,0,0,0,0,0,1,1,0,0,0,0,0,0,0,0,0,0,0,0,0,0,0,0,0,0,0,1,1,1,0,2,0,0,0,0,0,1,0,0,0,0,0,0,0,0,0,1,0,0,0,0,1,0,0,0,0,0,0,0,0,0,0,0,0,0,0,0,0,0),

.Dim=c(36,148)))

# Model for assessing host preference of root-associated fungi based on plant × fungal matrix in which plant OTUs were clustered with similarity cut-off of 99% (Data S4b)

model

{

for (i in 1:36)

{

pi[i,1:63]~ ddirch(alpha[i,]) # Dirichlet distribution

Y[i,1:63]~ dmulti(pi[i,],N[i]) # Multinomial distribution

D[i]<- sqrt(sum(d2[i,])) # Euclidean distance between empirical data vector and hypothetical data vector

for (j in 1:63)

{

alpha[i,j]<- T[j]*N[i]/442 # The expected number of host-fungus association under the random host selection hypothesis

d[i,j]<- pi[i,j]-alpha[i,j]/sum(alpha[i,])

d2[i,j]<- d[i,j]*d[i,j]

Dbar[i,j]<- d[i,j]/D[i] # Index of host preference

}

}

}

list(E=c(8,4,1,1,1,2,7,2,1,1,15,1,1,2,1,1,3,1,1,2,3,2,2,1,2,4,6,4,1,7,5,6,10,2,20,13,1,23,1,3,4,3,14,36,3,8,9,4,13,134,1,5,1,1,9,1,1,9,2,1,1,7,3),

N=c(115,112,107,103,89,85,77,69,60,56,56,52,49,48,48,47,47,46,40,39,37,37,34,31,28,26,22,17,15,14,13,13,12,11,11,11),

Y=structure(.Data=c(2,1,1,0,0,0,1,1,0,0,5,1,0,0,1,1,0,0,0,0,0,0,0,1,0,2,0,2,0,2,2,2,4,0,7,3,1,2,1,2,1,1,2,4,1,2,3,1,3,42,1,3,0,1,2,0,0,1,0,0,0,1,1,

2,1,1,0,0,0,2,0,0,0,6,0,0,1,0,0,1,0,1,1,1,0,0,1,1,0,3,0,0,1,0,0,0,0,9,3,1,1,0,0,0,0,2,6,1,1,2,0,6,44,0,3,0,0,2,0,0,4,0,1,0,2,1,

1,1,0,0,0,0,2,0,0,0,4,1,0,1,0,0,1,0,1,0,0,0,1,1,1,1,0,2,0,0,1,4,1,1,5,2,1,2,1,0,1,1,6,7,1,1,2,1,5,35,0,2,0,0,2,1,0,1,0,0,0,3,2,

0,2,0,0,0,1,0,0,1,1,2,0,0,1,0,0,2,0,0,0,1,1,0,0,1,1,3,0,1,2,5,0,4,0,8,0,0,4,0,0,1,0,5,10,2,2,0,0,3,21,1,0,0,0,4,0,0,6,1,0,1,4,1,

1,1,1,0,0,0,1,0,1,0,5,0,0,0,0,0,0,0,0,0,0,0,0,1,0,0,0,2,0,1,0,3,1,0,4,3,1,4,0,0,0,1,4,6,1,0,2,2,4,31,1,1,0,0,2,0,0,2,0,0,0,0,2,

1,2,0,0,0,2,0,0,1,1,1,0,1,0,1,0,0,0,0,0,0,2,0,0,0,0,1,1,1,2,3,0,7,2,4,6,0,2,0,0,0,0,1,4,0,0,0,1,4,28,0,0,0,0,2,0,0,2,0,0,0,2,0,

3,0,0,0,0,0,1,0,0,0,0,0,0,0,0,0,0,0,0,0,1,0,0,1,1,2,0,1,1,1,0,1,1,0,5,3,0,0,1,1,2,1,3,2,0,1,1,1,2,27,1,1,1,0,1,0,0,4,0,1,1,2,1,

2,1,0,0,0,0,4,0,0,0,0,0,0,1,0,0,0,0,1,2,1,0,0,0,1,2,4,0,0,3,1,1,0,0,9,0,0,0,0,1,1,1,4,18,0,0,0,0,0,5,0,1,0,0,3,0,1,1,0,0,0,0,0,

0,0,1,0,0,0,1,2,0,0,5,0,0,0,0,0,0,0,0,0,1,0,0,1,0,1,1,0,0,0,0,0,0,0,5,0,0,4,0,0,0,0,1,4,0,1,2,0,2,18,0,0,0,0,3,0,0,1,0,0,0,3,3,

0,1,0,0,0,0,0,0,1,0,2,0,0,0,1,0,0,0,0,0,0,1,0,0,0,0,1,1,0,2,1,0,3,1,0,5,0,0,0,1,0,0,1,3,0,0,0,1,1,22,0,0,0,0,4,0,0,1,0,0,0,2,0,

1,0,0,0,0,1,2,0,0,0,0,0,0,0,0,0,0,1,1,0,0,0,2,1,0,1,1,1,0,1,0,1,2,0,1,5,0,2,0,0,0,0,2,0,0,0,0,1,2,23,0,1,0,0,0,0,0,2,0,0,0,1,0,

2,3,0,0,0,0,1,0,0,0,1,0,0,0,0,0,1,0,1,0,0,0,0,1,0,0,0,2,0,2,0,0,0,0,5,2,0,1,0,0,0,0,1,4,1,0,2,0,1,17,0,0,0,1,0,0,0,2,0,1,0,0,0,

1,1,0,0,0,1,0,1,0,1,0,0,0,1,0,0,1,0,0,0,1,1,0,0,1,1,2,0,1,1,0,0,1,0,2,0,0,1,0,0,0,0,3,3,1,1,1,0,0,9,1,0,0,0,2,0,0,3,1,0,0,4,1,

1,0,0,0,0,0,0,0,0,0,9,0,0,0,0,0,1,0,0,0,0,0,1,0,0,0,0,0,0,0,1,0,0,0,0,0,0,0,0,0,0,0,1,0,0,0,0,0,4,25,0,1,0,0,0,0,0,2,0,0,1,0,1,

0,1,0,0,0,0,1,0,0,1,0,0,0,1,0,0,0,0,0,0,1,0,0,0,0,0,2,0,0,3,0,0,1,1,3,1,0,2,0,0,1,1,2,2,1,0,0,0,2,15,0,0,0,0,2,0,0,3,0,0,0,1,0,

1,0,0,0,0,0,1,0,0,0,0,0,0,0,1,0,0,0,0,0,1,1,0,1,0,2,0,1,0,0,0,5,0,0,2,1,0,0,0,0,0,0,6,3,0,2,2,0,0,11,0,2,1,1,1,0,0,1,0,0,0,0,0,

1,0,1,0,0,0,1,0,0,0,3,0,0,1,0,1,0,0,0,0,0,0,0,0,1,0,1,0,0,2,1,0,0,0,3,0,0,1,0,0,0,1,1,1,2,0,1,1,2,18,0,0,0,0,1,0,0,0,0,1,0,1,0,

0,1,0,1,0,0,0,0,1,0,0,0,1,0,0,0,0,0,1,0,0,1,0,1,0,0,1,1,0,1,1,3,1,0,2,4,1,1,0,0,0,0,2,5,1,0,0,0,1,6,1,1,1,1,1,0,0,2,0,0,1,0,0,

0,1,0,0,0,0,2,0,0,0,0,0,0,0,0,0,0,0,0,0,0,0,0,0,0,1,0,0,0,0,0,0,1,0,3,2,0,1,0,0,0,0,3,2,1,0,0,2,1,17,0,0,0,0,0,0,0,1,0,0,0,1,1,

0,1,1,0,0,0,1,0,0,0,1,0,0,0,0,0,0,1,0,0,0,1,1,0,0,1,0,0,0,1,0,0,1,0,3,2,0,0,0,0,1,0,1,4,1,0,0,0,0,10,0,0,0,0,2,1,0,1,0,1,1,1,0,

0,1,1,0,0,1,2,0,0,0,1,0,0,0,0,0,0,0,0,1,0,0,0,0,0,3,0,0,0,0,0,0,0,1,2,2,0,0,0,0,1,0,1,7,1,0,0,0,0,5,0,2,0,0,1,1,0,0,0,1,1,1,0,

0,1,0,0,0,0,1,1,0,1,0,0,0,0,0,0,0,1,1,0,2,1,0,0,0,0,1,0,0,0,1,0,0,0,1,2,0,4,0,0,2,0,1,4,0,2,0,0,0,9,0,0,0,0,0,0,0,0,0,0,0,0,1,

0,1,0,1,0,1,1,0,0,0,0,0,0,0,0,0,0,1,0,0,1,0,0,0,1,1,0,1,0,0,0,0,0,0,5,0,0,1,0,0,1,0,0,2,0,2,1,0,1,11,0,0,0,0,0,0,0,1,0,0,0,0,0,

0,0,0,0,0,0,0,0,0,0,0,0,0,0,0,0,0,0,1,0,0,0,0,0,0,0,1,0,0,0,0,5,0,0,4,1,1,0,1,2,0,0,0,3,0,0,1,1,0,6,0,1,1,1,0,0,0,1,0,0,0,0,0,

0,0,0,0,0,0,0,0,0,0,0,1,0,0,0,0,0,0,0,0,0,0,0,0,0,0,0,0,0,0,0,0,0,0,0,0,0,1,1,0,0,0,1,0,0,0,0,0,0,23,0,0,0,0,0,0,0,0,0,0,0,1,0,

0,0,0,0,0,0,0,0,0,0,0,0,0,0,0,1,0,0,0,0,0,0,0,1,0,0,1,0,0,0,0,0,1,0,1,2,0,0,0,0,0,0,0,0,1,0,1,1,1,15,0,0,0,0,0,0,0,0,0,0,0,0,0,

0,0,0,0,1,0,1,0,0,0,1,0,0,0,0,0,0,0,0,0,0,0,0,0,0,0,0,2,0,1,0,1,1,0,0,0,0,1,0,0,0,0,0,1,0,0,0,1,0,9,0,1,0,0,0,0,0,0,0,0,0,1,0,

0,0,0,0,0,0,0,0,0,0,0,0,0,0,0,0,0,0,0,0,0,0,0,0,0,0,0,0,0,0,0,0,0,0,0,0,0,0,0,0,0,0,0,0,0,0,7,0,0,10,0,0,0,0,0,0,0,0,0,0,0,0,0,

0,0,0,0,0,0,0,0,0,0,0,0,0,0,0,0,0,0,1,0,0,0,0,0,0,0,0,0,0,0,0,0,0,0,1,0,0,1,0,0,0,0,0,1,0,0,0,0,0,7,0,1,0,0,1,0,0,1,0,1,0,0,0,

0,0,0,0,0,0,0,0,0,0,2,0,0,0,0,0,0,0,0,0,0,0,0,0,0,0,0,0,0,0,0,0,0,0,2,0,0,0,0,0,0,0,0,0,0,0,0,0,5,4,0,0,1,0,0,0,0,0,0,0,0,0,0,

0,0,0,0,0,0,0,0,0,0,0,0,0,0,0,0,0,0,0,0,0,0,0,0,0,0,0,0,0,0,0,0,0,0,1,0,0,0,0,0,0,0,0,0,0,0,0,0,0,11,0,1,0,0,0,0,0,0,0,0,0,0,0,

0,0,0,0,0,0,0,0,0,0,0,0,0,0,0,0,0,0,0,0,0,0,0,0,0,0,0,0,0,0,0,0,0,0,0,1,0,2,0,0,0,0,1,1,0,1,0,0,0,5,0,0,0,1,0,0,0,1,0,0,0,0,0,

0,0,0,0,0,0,0,0,0,0,3,0,0,0,0,0,0,0,0,0,0,0,0,0,0,0,0,0,0,1,0,0,0,0,0,0,0,0,0,0,0,0,0,0,0,0,0,1,1,6,0,0,0,0,0,0,0,0,0,0,0,0,0,

0,0,0,0,0,0,0,0,0,0,1,0,0,0,1,0,0,0,0,0,0,0,0,0,0,1,0,0,0,0,0,0,1,0,0,0,0,0,0,1,0,0,0,0,0,0,0,0,0,5,0,0,0,0,1,0,0,0,0,0,0,0,0,

0,0,0,0,0,0,0,0,0,0,1,0,0,0,0,0,0,0,0,0,0,0,0,0,0,0,0,0,1,0,0,0,0,0,0,1,0,0,0,0,0,0,0,1,0,0,1,0,0,5,0,0,0,0,1,0,0,0,0,0,0,0,0,

0,0,0,0,0,0,0,0,0,0,2,0,0,0,0,0,0,0,0,0,0,0,0,0,0,0,0,0,0,0,0,0,0,0,0,0,0,1,0,0,0,0,0,2,0,0,0,0,0,6,0,0,0,0,0,0,0,0,0,0,0,0,0),

.Dim=c(36,63)))

# Model for assessing host preference of root-associated fungi based on plant × fungal matrix in which plant OTUs were clustered with similarity cut-off of 98% (Data S4c)

model

{

for (i in 1:36)

{

pi[i,1:52]~ ddirch(alpha[i,]) # Dirichlet distribution

Y[i,1:52]~ dmulti(pi[i,],N[i]) # Multinomial distribution

D[i]<- sqrt(sum(d2[i,])) # Euclidean distance between empirical data vector and hypothetical data vector

for (j in 1:52)

{

alpha[i,j]<- T[j]*N[i]/442 # The expected number of host-fungus association under the random host selection hypothesis

d[i,j]<- pi[i,j]-alpha[i,j]/sum(alpha[i,])

d2[i,j]<- d[i,j]*d[i,j]

Dbar[i,j]<- d[i,j]/D[i] # Index of host preference

}

}

}

list(E=c(13,1,1,2,9,1,1,15,1,1,2,1,1,3,1,1,2,3,2,2,1,2,4,6,5,7,5,6,10,22,14,23,1,3,4,3,14,39,8,9,17,134,1,16,1,1,9,2,1,1,7,3),

N=c(115,112,107,103,89,85,77,69,60,56,56,52,49,48,48,47,47,46,40,39,37,37,34,31,28,26,22,17,15,14,13,13,12,11,11,11),

Y=structure(.Data=c(4,0,0,0,2,0,0,5,1,0,0,1,1,0,0,0,0,0,0,0,1,0,2,0,2,2,2,2,4,7,4,2,1,2,1,1,2,5,2,3,4,42,1,6,0,0,1,0,0,0,1,1,

4,0,0,0,2,0,0,6,0,0,1,0,0,1,0,1,1,1,0,0,1,1,0,3,0,1,0,0,0,9,4,1,0,0,0,0,2,7,1,2,6,44,0,5,0,0,4,0,1,0,2,1,

2,0,0,0,2,0,0,4,1,0,1,0,0,1,0,1,0,0,0,1,1,1,1,0,2,0,1,4,1,6,3,2,1,0,1,1,6,8,1,2,6,35,0,4,1,0,1,0,0,0,3,2,

2,0,0,1,0,1,1,2,0,0,1,0,0,2,0,0,0,1,1,0,0,1,1,3,1,2,5,0,4,8,0,4,0,0,1,0,5,12,2,0,3,21,1,4,0,0,6,1,0,1,4,1,

3,0,0,0,1,1,0,5,0,0,0,0,0,0,0,0,0,0,0,0,1,0,0,0,2,1,0,3,1,4,4,4,0,0,0,1,4,7,0,2,6,31,1,3,0,0,2,0,0,0,0,2,

3,0,0,2,0,1,1,1,0,1,0,1,0,0,0,0,0,0,2,0,0,0,0,1,2,2,3,0,7,6,6,2,0,0,0,0,1,4,0,0,5,28,0,2,0,0,2,0,0,0,2,0,

3,0,0,0,1,0,0,0,0,0,0,0,0,0,0,0,0,1,0,0,1,1,2,0,2,1,0,1,1,5,3,0,1,1,2,1,3,2,1,1,3,27,1,3,0,0,4,0,1,1,2,1,

3,0,0,0,4,0,0,0,0,0,1,0,0,0,0,1,2,1,0,0,0,1,2,4,0,3,1,1,0,9,0,0,0,1,1,1,4,18,0,0,0,5,0,4,0,1,1,0,0,0,0,0,

1,0,0,0,3,0,0,5,0,0,0,0,0,0,0,0,0,1,0,0,1,0,1,1,0,0,0,0,0,5,0,4,0,0,0,0,1,4,1,2,2,18,0,3,0,0,1,0,0,0,3,3,

1,0,0,0,0,1,0,2,0,0,0,1,0,0,0,0,0,0,1,0,0,0,0,1,1,2,1,0,3,1,5,0,0,1,0,0,1,3,0,0,2,22,0,4,0,0,1,0,0,0,2,0,

1,0,0,1,2,0,0,0,0,0,0,0,0,0,1,1,0,0,0,2,1,0,1,1,1,1,0,1,2,1,5,2,0,0,0,0,2,0,0,0,3,23,0,1,0,0,2,0,0,0,1,0,

5,0,0,0,1,0,0,1,0,0,0,0,0,1,0,1,0,0,0,0,1,0,0,0,2,2,0,0,0,5,2,1,0,0,0,0,1,5,0,2,1,17,0,1,0,0,2,0,1,0,0,0,

2,0,0,1,1,0,1,0,0,0,1,0,0,1,0,0,0,1,1,0,0,1,1,2,1,1,0,0,1,2,0,1,0,0,0,0,3,4,1,1,0,9,1,2,0,0,3,1,0,0,4,1,

1,0,0,0,0,0,0,9,0,0,0,0,0,1,0,0,0,0,0,1,0,0,0,0,0,0,1,0,0,0,0,0,0,0,0,0,1,0,0,0,4,25,0,1,0,0,2,0,0,1,0,1,

1,0,0,0,1,0,1,0,0,0,1,0,0,0,0,0,0,1,0,0,0,0,0,2,0,3,0,0,1,4,1,2,0,0,1,1,2,3,0,0,2,15,0,2,0,0,3,0,0,0,1,0,

1,0,0,0,1,0,0,0,0,0,0,1,0,0,0,0,0,1,1,0,1,0,2,0,1,0,0,5,0,2,1,0,0,0,0,0,6,3,2,2,0,11,0,5,0,0,1,0,0,0,0,0,

2,0,0,0,1,0,0,3,0,0,1,0,1,0,0,0,0,0,0,0,0,1,0,1,0,2,1,0,0,3,0,1,0,0,0,1,1,3,0,1,3,18,0,1,0,0,0,0,1,0,1,0,

1,1,0,0,0,1,0,0,0,1,0,0,0,0,0,1,0,0,1,0,1,0,0,1,1,1,1,3,1,2,5,1,0,0,0,0,2,6,0,0,1,6,1,4,0,0,2,0,0,1,0,0,

1,0,0,0,2,0,0,0,0,0,0,0,0,0,0,0,0,0,0,0,0,0,1,0,0,0,0,0,1,3,2,1,0,0,0,0,3,3,0,0,3,17,0,0,0,0,1,0,0,0,1,1,

2,0,0,0,1,0,0,1,0,0,0,0,0,0,1,0,0,0,1,1,0,0,1,0,0,1,0,0,1,3,2,0,0,0,1,0,1,5,0,0,0,10,0,2,1,0,1,0,1,1,1,0,

2,0,0,1,2,0,0,1,0,0,0,0,0,0,0,0,1,0,0,0,0,0,3,0,0,0,0,0,0,3,2,0,0,0,1,0,1,8,0,0,0,5,0,3,1,0,0,0,1,1,1,0,

1,0,0,0,2,0,1,0,0,0,0,0,0,0,1,1,0,2,1,0,0,0,0,1,0,0,1,0,0,1,2,4,0,0,2,0,1,4,2,0,0,9,0,0,0,0,0,0,0,0,0,1,

1,1,0,1,1,0,0,0,0,0,0,0,0,0,1,0,0,1,0,0,0,1,1,0,1,0,0,0,0,5,0,1,0,0,1,0,0,2,2,1,1,11,0,0,0,0,1,0,0,0,0,0,

0,0,0,0,0,0,0,0,0,0,0,0,0,0,0,1,0,0,0,0,0,0,0,1,0,0,0,5,0,4,2,0,1,2,0,0,0,3,0,1,1,6,0,3,0,0,1,0,0,0,0,0,

0,0,0,0,0,0,0,0,1,0,0,0,0,0,0,0,0,0,0,0,0,0,0,0,0,0,0,0,0,0,0,1,1,0,0,0,1,0,0,0,0,23,0,0,0,0,0,0,0,0,1,0,

0,0,0,0,0,0,0,0,0,0,0,0,1,0,0,0,0,0,0,0,1,0,0,1,0,0,0,0,1,1,2,0,0,0,0,0,0,1,0,1,2,15,0,0,0,0,0,0,0,0,0,0,

0,0,1,0,1,0,0,1,0,0,0,0,0,0,0,0,0,0,0,0,0,0,0,0,2,1,0,1,1,0,0,1,0,0,0,0,0,1,0,0,1,9,0,1,0,0,0,0,0,0,1,0,

0,0,0,0,0,0,0,0,0,0,0,0,0,0,0,0,0,0,0,0,0,0,0,0,0,0,0,0,0,0,0,0,0,0,0,0,0,0,0,7,0,10,0,0,0,0,0,0,0,0,0,0,

0,0,0,0,0,0,0,0,0,0,0,0,0,0,0,1,0,0,0,0,0,0,0,0,0,0,0,0,0,1,0,1,0,0,0,0,0,1,0,0,0,7,0,2,0,0,1,0,1,0,0,0,

0,0,0,0,0,0,0,2,0,0,0,0,0,0,0,0,0,0,0,0,0,0,0,0,0,0,0,0,0,2,0,0,0,0,0,0,0,0,0,0,5,4,0,1,0,0,0,0,0,0,0,0,

0,0,0,0,0,0,0,0,0,0,0,0,0,0,0,0,0,0,0,0,0,0,0,0,0,0,0,0,0,1,0,0,0,0,0,0,0,0,0,0,0,11,0,1,0,0,0,0,0,0,0,0,

0,0,0,0,0,0,0,0,0,0,0,0,0,0,0,0,0,0,0,0,0,0,0,0,0,0,0,0,0,0,1,2,0,0,0,0,1,1,1,0,0,5,0,1,0,0,1,0,0,0,0,0,

0,0,0,0,0,0,0,3,0,0,0,0,0,0,0,0,0,0,0,0,0,0,0,0,0,1,0,0,0,0,0,0,0,0,0,0,0,0,0,0,2,6,0,0,0,0,0,0,0,0,0,0,

0,0,0,0,0,0,0,1,0,0,0,1,0,0,0,0,0,0,0,0,0,0,1,0,0,0,0,0,1,0,0,0,0,1,0,0,0,0,0,0,0,5,0,1,0,0,0,0,0,0,0,0,

0,0,0,0,0,0,0,1,0,0,0,0,0,0,0,0,0,0,0,0,0,0,0,0,1,0,0,0,0,0,1,0,0,0,0,0,0,1,0,1,0,5,0,1,0,0,0,0,0,0,0,0,

0,0,0,0,0,0,0,2,0,0,0,0,0,0,0,0,0,0,0,0,0,0,0,0,0,0,0,0,0,0,0,1,0,0,0,0,0,2,0,0,0,6,0,0,0,0,0,0,0,0,0,0),

.Dim=c(36,52)))

# Model for assessing host preference of root-associated fungi based on plant × fungal matrix in which plant OTUs were clustered with similarity cut-off of 97% (Data S4d)

model

{

for (i in 1:36)

{

pi[i,1:39]~ ddirch(alpha[i,]) # Dirichlet distribution

Y[i,1:39]~ dmulti(pi[i,],N[i]) # Multinomial distribution

D[i]<- sqrt(sum(d2[i,])) # Euclidean distance between empirical data vector and hypothetical data vector

for (j in 1:39)

{

alpha[i,j]<- T[j]*N[i]/442 # The expected number of host-fungus association under the random host selection hypothesis

d[i,j]<- pi[i,j]-alpha[i,j]/sum(alpha[i,])

d2[i,j]<- d[i,j]*d[i,j]

Dbar[i,j]<- d[i,j]/D[i] # Index of host preference

}

}

}

list(E=c(14,1,2,9,1,1,2,2,2,1,1,3,1,1,2,5,2,6,6,5,1,5,6,10,22,14,40,14,39,8,174,1,18,9,2,1,1,7,3),

N=c(115,112,107,103,89,85,77,69,60,56,56,52,49,48,48,47,47,46,40,39,37,37,34,31,28,26,22,17,15,14,13,13,12,11,11,11),

Y=structure(.Data=c(4,0,0,2,0,0,1,1,0,1,1,0,0,0,0,0,0,2,0,2,0,2,2,4,7,4,9,2,5,2,54,1,6,1,0,0,0,1,1,

4,0,0,2,0,0,1,0,1,0,0,1,0,1,1,1,0,1,3,0,0,0,0,0,9,4,2,2,7,1,58,0,5,4,0,1,0,2,1,

2,0,0,2,0,0,1,1,1,0,0,1,0,1,0,0,1,2,0,2,0,1,4,1,6,3,5,6,8,1,47,0,5,1,0,0,0,3,2,

2,0,1,0,1,1,0,0,1,0,0,2,0,0,0,2,0,2,3,1,0,5,0,4,8,0,7,5,12,2,26,1,4,6,1,0,1,4,1,

3,0,0,1,1,0,1,0,0,0,0,0,0,0,0,0,0,0,0,2,0,0,3,1,4,4,6,4,7,0,44,1,3,2,0,0,0,0,2,

3,0,2,0,1,1,0,1,0,1,0,0,0,0,0,2,0,0,1,2,0,3,0,7,6,6,4,1,4,0,34,0,2,2,0,0,0,2,0,

3,0,0,1,0,0,0,0,0,0,0,0,0,0,0,1,0,3,0,2,0,0,1,1,5,3,6,3,2,1,32,1,3,4,0,1,1,2,1,

3,0,0,4,0,0,0,0,1,0,0,0,0,1,2,1,0,3,4,0,1,1,1,0,9,0,5,4,18,0,5,0,5,1,0,0,0,0,0,

1,0,0,3,0,0,1,0,0,0,0,0,0,0,0,1,0,1,1,0,0,0,0,0,5,0,4,1,4,1,27,0,3,1,0,0,0,3,3,

1,0,0,0,1,0,0,0,0,1,0,0,0,0,0,1,0,0,1,1,0,1,0,3,1,5,3,1,3,0,26,0,4,1,0,0,0,2,0,

1,0,1,2,0,0,0,0,0,0,0,0,1,1,0,0,2,1,1,1,0,0,1,2,1,5,3,2,0,0,27,0,1,2,0,0,0,1,0,

5,0,0,1,0,0,0,0,0,0,0,1,0,1,0,0,0,0,0,2,0,0,0,0,5,2,3,1,5,0,22,0,1,2,0,1,0,0,0,

2,0,1,1,0,1,0,0,1,0,0,1,0,0,0,2,0,2,2,1,0,0,0,1,2,0,2,3,4,1,10,1,2,3,1,0,0,4,1,

1,0,0,0,0,0,1,0,0,0,0,1,0,0,0,0,1,0,0,0,0,1,0,0,0,0,0,1,0,0,37,0,1,2,0,0,1,0,1,

1,0,0,1,0,1,0,0,1,0,0,0,0,0,0,1,0,0,2,0,1,0,0,1,4,1,6,2,3,0,17,0,2,3,0,0,0,1,0,

1,0,0,1,0,0,0,0,0,1,0,0,0,0,0,2,0,2,0,1,0,0,5,0,2,1,0,6,3,2,14,0,5,1,0,0,0,0,0,

2,0,0,1,0,0,1,0,1,0,1,0,0,0,0,0,0,1,1,0,1,1,0,0,3,0,3,1,3,0,24,0,1,0,0,1,0,1,0,

2,0,0,0,1,0,0,1,0,0,0,0,0,1,0,1,0,0,1,1,0,1,3,1,2,5,2,2,6,0,8,1,4,2,0,0,1,0,0,

1,0,0,2,0,0,0,0,0,0,0,0,0,0,0,0,0,1,0,0,0,0,0,1,3,2,1,3,3,0,20,0,0,1,0,0,0,1,1,

2,0,0,1,0,0,0,0,0,0,0,0,1,0,0,1,1,1,0,0,0,0,0,1,3,2,2,1,5,0,11,0,3,1,0,1,1,1,0,

2,0,1,2,0,0,0,0,0,0,0,0,0,0,1,0,0,3,0,0,0,0,0,0,3,2,1,1,8,0,6,0,4,0,0,1,1,1,0,

1,0,0,2,0,1,0,0,0,0,0,0,1,1,0,3,0,0,1,0,0,1,0,0,1,2,6,1,4,2,9,0,0,0,0,0,0,0,1,

2,0,1,1,0,0,0,0,0,0,0,0,1,0,0,1,0,2,0,1,0,0,0,0,5,0,2,0,2,2,13,0,0,1,0,0,0,0,0,

0,0,0,0,0,0,0,0,0,0,0,0,0,1,0,0,0,0,1,0,0,0,5,0,4,2,3,0,3,0,8,0,3,1,0,0,0,0,0,

0,0,0,0,0,0,0,1,0,0,0,0,0,0,0,0,0,0,0,0,0,0,0,0,0,0,2,1,0,0,23,0,0,0,0,0,0,1,0,

0,0,0,0,0,0,0,0,0,0,1,0,0,0,0,0,0,0,1,0,0,0,0,1,1,2,0,0,1,0,19,0,0,0,0,0,0,0,0,

0,1,0,1,0,0,0,0,0,0,0,0,0,0,0,0,0,0,0,2,0,0,1,1,0,0,2,0,1,0,11,0,1,0,0,0,0,1,0,

0,0,0,0,0,0,0,0,0,0,0,0,0,0,0,0,0,0,0,0,0,0,0,0,0,0,0,0,0,0,17,0,0,0,0,0,0,0,0,

0,0,0,0,0,0,0,0,0,0,0,0,0,1,0,0,0,0,0,0,0,0,0,0,1,0,1,0,1,0,7,0,2,1,0,1,0,0,0,

0,0,0,0,0,0,0,0,0,0,0,0,0,0,0,0,0,0,0,0,0,0,0,0,2,0,0,0,0,0,11,0,1,0,0,0,0,0,0,

0,0,0,0,0,0,0,0,0,0,0,0,0,0,0,0,0,0,0,0,0,0,0,0,1,0,0,0,0,0,11,0,1,0,0,0,0,0,0,

0,0,0,0,0,0,0,0,0,0,0,0,0,0,0,0,0,0,0,0,0,0,0,0,0,1,2,1,1,1,5,0,1,1,0,0,0,0,0,

0,0,0,0,0,0,1,0,0,0,0,0,0,0,0,0,0,0,0,0,0,0,0,0,0,0,1,0,0,0,10,0,0,0,0,0,0,0,0,

0,0,0,0,0,0,0,0,0,1,0,0,0,0,0,0,0,1,0,0,0,0,0,1,0,0,1,0,0,0,6,0,1,0,0,0,0,0,0,

0,0,0,0,0,0,1,0,0,0,0,0,0,0,0,0,0,0,0,1,0,0,0,0,0,1,0,0,1,0,6,0,1,0,0,0,0,0,0,

0,0,0,0,0,0,0,0,0,0,0,0,0,0,0,0,0,0,0,0,0,0,0,0,0,0,1,0,2,0,8,0,0,0,0,0,0,0,0),

.Dim=c(36,39)))
