# Supplementary material for: Contrasting Diversity and Host Association of Ectomycorrhizal Basidiomycetes versus Root-Associated Ascomycetes in a Dipterocarp Rainforest
Source: PLoS One. 2015 Apr 17;10(4):e0125550. doi: 10.1371/journal.pone.0125550 (PMC4401655; doi:10.1371/journal.pone.0125550)
Supplement: S1 Table — Mantel tests were performed for dipterocarp (dip) datasets and non-dipterocarp (non-dip) datasets, separately. In partial Mantel tests, correlation between the two variables was tested after controlling for the effect of genetic distance of host plants. (DOCX) [file pone.0125550.s014.docx]

**S1_Table** Results of standard and partial Mantel tests between community dissimilarity of fungi and geographic distance. Mantel tests were performed for dipterocarp (dip) datasets and non-dipterocarp (non-dip) datasets, separately. In partial Mantel test, correlation between two variables were tested after controlling for the effect of genetic distance of host plants.

| **Taxa** | **Dataset** | **Statistical analysis** | **Mantel r** | **P value** |
| --- | --- | --- | --- | --- |
| All fungal taxa | dip | Standard Mantel | 0.028 | 0.019 |
|  | dip | Partial Mantel | 0.027 | 0.028 |
|  | non-dip | Standard Mantel | 0.044 | 0.002 |
|  | non-dip | Partial Mantel | 0.044 | 0.002 |
| Ascomycota | dip | Standard Mantel | 0.015 | 0.132 |
|  | dip | Partial Mantel | 0.015 | 0.136 |
|  | non-dip | Standard Mantel | 0.032 | 0.007 |
|  | non-dip | Partial Mantel | 0.031 | 0.009 |
| Basidiomycota | dip | Standard Mantel | 0.039 | <0.00１ |
|  | dip | Partial Mantel | 0.041 | <0.00１ |
|  | non-dip | Standard Mantel | 0.030 | 0.008 |
|  | non-dip | Partial Mantel | 0.028 | 0.012 |
| ECM Basidiomycota | dip | Standard Mantel | 0.037 | 0.002 |
|  | dip | Partial Mantel | 0.039 | 0.001 |
|  | non-dip | Standard Mantel | 0.046 | 0.002 |
|  | non-dip | Partial Mantel | 0.047 | 0.001 |
| Basidiomycota | dip | Standard Mantel | 0.030 | 0.004 |
| (Excluding ECM taxa) | dip | Partial Mantel | 0.030 | 0.005 |
|  | non-dip | Standard Mantel | 0.020 | 0.053 |
|  | non-dip | Partial Mantel | 0.019 | 0.073 |
